# Pharmacological conversion of gut epithelial cells into insulin-producing cells lowers glycemia in diabetic animals

**DOI:** 10.1172/JCI162720

**Published:** 2022-12-15

**Authors:** Wen Du, Junqiang Wang, Taiyi Kuo, Liheng Wang, Wendy M. McKimpson, Jinsook Son, Hitoshi Watanabe, Takumi Kitamoto, Yunkyoung Lee, Remi J. Creusot, Lloyd E. Ratner, Kasi McCune, Ya-Wen Chen, Brendan H. Grubbs, Matthew E. Thornton, Jason Fan, Nishat Sultana, Bryan S. Diaz, Iyshwarya Balasubramanian, Nan Gao, Sandro Belvedere, Domenico Accili

**Affiliations:** 1 Department of Medicine and Naomi Berrie Diabetes Center and; 2 Systems Biology Institute, Vagelos College of Physicians and Surgeons, Columbia University, New York, New York, USA.; 3 Department of Neurobiology, Physiology, & Behavior, College of Biological Sciences, University of California, Davis, California, USA.; 4 Forkhead BioTherapeutics Corp., New York, New York, USA.; 5 Columbia Center for Translational Immunology, Department of Medicine, Columbia University Irving Medical Center, New York, New York, USA.; 6 Department of Surgery, Columbia University Medical Center, New York, New York, USA.; 7 Department of Otolaryngology,; 8 Department of Cell, Developmental, and Regenerative Biology, and; 9 Black Family Stem Cell Institute, Icahn School of Medicine at Mount Sinai, New York, New York, USA.; 10 Department of Obstetrics and Gynecology, Keck School of Medicine, University of Southern California, Los Angeles, California, USA.; 11 Bascom Palmer Eye Institute, Department of Ophthalmology, Miami, Florida, USA.; 12 Department of Biological Sciences, Rutgers University, Newark, New Jersey, USA.

**Keywords:** Endocrinology, Beta cells, Diabetes, Insulin

## Abstract

As a highly regenerative organ, the intestine is a promising source for cellular reprogramming for replacing lost pancreatic **β** cells in diabetes. Gut enterochromaffin cells can be converted to insulin-producing cells by forkhead box O1 (*FoxO1*) ablation, but their numbers are limited. In this study, we report that insulin-immunoreactive cells with Paneth/goblet cell features are present in human fetal intestine. Accordingly, lineage-tracing experiments show that, upon genetic or pharmacologic FoxO1 ablation, the Paneth/goblet lineage can also undergo conversion to the insulin lineage. We designed a screening platform in gut organoids to accurately quantitate **β**-like cell reprogramming and fine-tune a combination treatment to increase the efficiency of the conversion process in mice and human adult intestinal organoids. We identified a triple blockade of FOXO1, Notch, and TGF-**β** that, when tested in insulin-deficient streptozotocin (STZ) or NOD diabetic animals, resulted in near normalization of glucose levels, associated with the generation of intestinal insulin-producing cells. The findings illustrate a therapeutic approach for replacing insulin treatment in diabetes.

## Graphical abstract

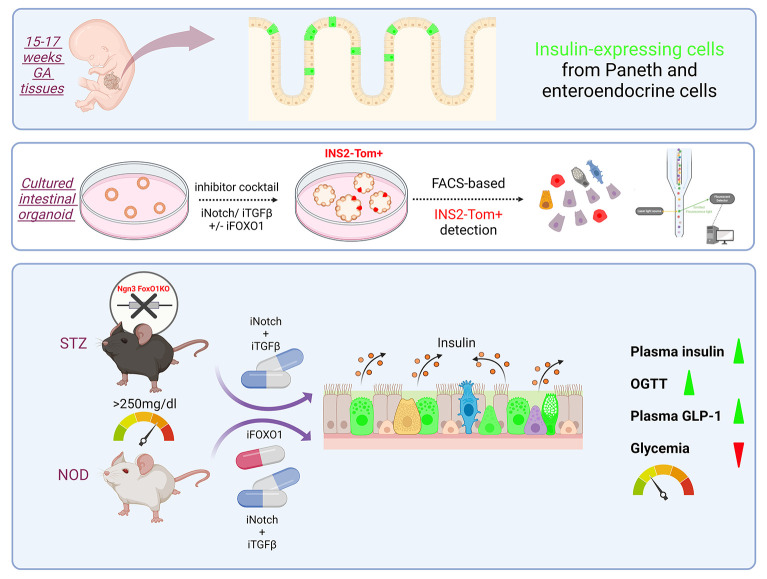

## Introduction

The CDC estimates that 1.6 million adults and 283,000 children and adolescents have type 1 diabetes (T1D) in the US alone (https://www.cdc.gov/diabetes/data/statistics-report/diagnosed-diabetes.html). Insulin replacement is a life-saving treatment, but it is not a cure and poses a substantial burden on patients and their families. Restoration of endogenous insulin production to cure T1D remains a topic of intense interest (1). Two alternative approaches have been proposed: transplantation and regeneration.

Beginning as early as the 1970s, isolated cadaveric pancreatic islets have been transplanted into T1D recipients to treat diabetes (2). Recent developments in stem cell technologies enabled human ES cell–derived (hESC-derived) or induced pluripotent stem cell–derived (iPSC-derived) islet replacement (3–5). Mature glucose-responsive β-like cells that are functionally equivalent to cadaveric islets can be obtained by different protocols (6–8). Two studies reporting interim data from ongoing first-in-human iPSC-based transplants showed restoration of meal-induced C-peptide response for up to 1 year in one patient after implantation of iPSC-derived islet cells, providing proof of concept for this approach (9, 10).

Another potential solution is to convert cell types developmentally related to pancreatic β cells into functional insulin-secreting cells in vivo. However, despite a wealth of reports illustrating conversion of different cell types into β-like cells, poor reproducibility has plagued this area, as has the intrinsic difficulty of targeting this process pharmacologically (11, 12). Although most studies focused on conversion of different pancreatic cell types, there are reports of transdifferentiation from organs developmentally related to the pancreas, such as liver (13, 14), stomach (15), and intestine (16).

Genetic ablation of forkhead box O1 (*FoxO1*) in neurogenin 3–positive (*Neurog3^+^*) progenitor cells can convert enteroendocrine cells (EEC) into insulin-producing β-like cells in mice (17). Moreover, FOXO1 inhibition using a dominant-negative mutant or lentivirus-encoded shRNA promotes generation of insulin-secreting cells in human iPSC–derived gut organoids (GOs) (18). The potential therapeutic significance of this work was amplified by recent reports: one identifying β-like cells in the human fetal intestine — and thus implying that conversion restores a fetal cell type (19); and others showing that previously described small molecule FOXO1 inhibitors can yield insulin-producing cells in vivo and lower glycemia in diabetic mice (20, 21). These findings prompted us to investigate whether other descendants of *Neurog3^+^* progenitors, such as subsets of goblet and Paneth cells, have the potential to be converted into insulin-secreting β-like cells. Based on the identification of cells with mixed lineage of insulin and Paneth/goblet features in human fetal intestines, we developed cellular assays to accurately quantitate cell reprogramming and sought to identify a combination treatment to increase the efficiency of the conversion process by leveraging the expansion of the Neurogenin 3 and Paneth/goblet lineages. We found that triple blockade of FOXO1, Notch, and TGF-β can bolster conversion and result in a robust glucose-lowering effect in streptozotocin and NOD diabetic animals. Our findings provide a mechanism underlying intestinal cell transdifferentiation into pancreatic β-like cells and expand its potential therapeutic applications.

## Results

### A subset of fetal insulin-positive intestinal secretory lineage cells.

**Figure 1:**
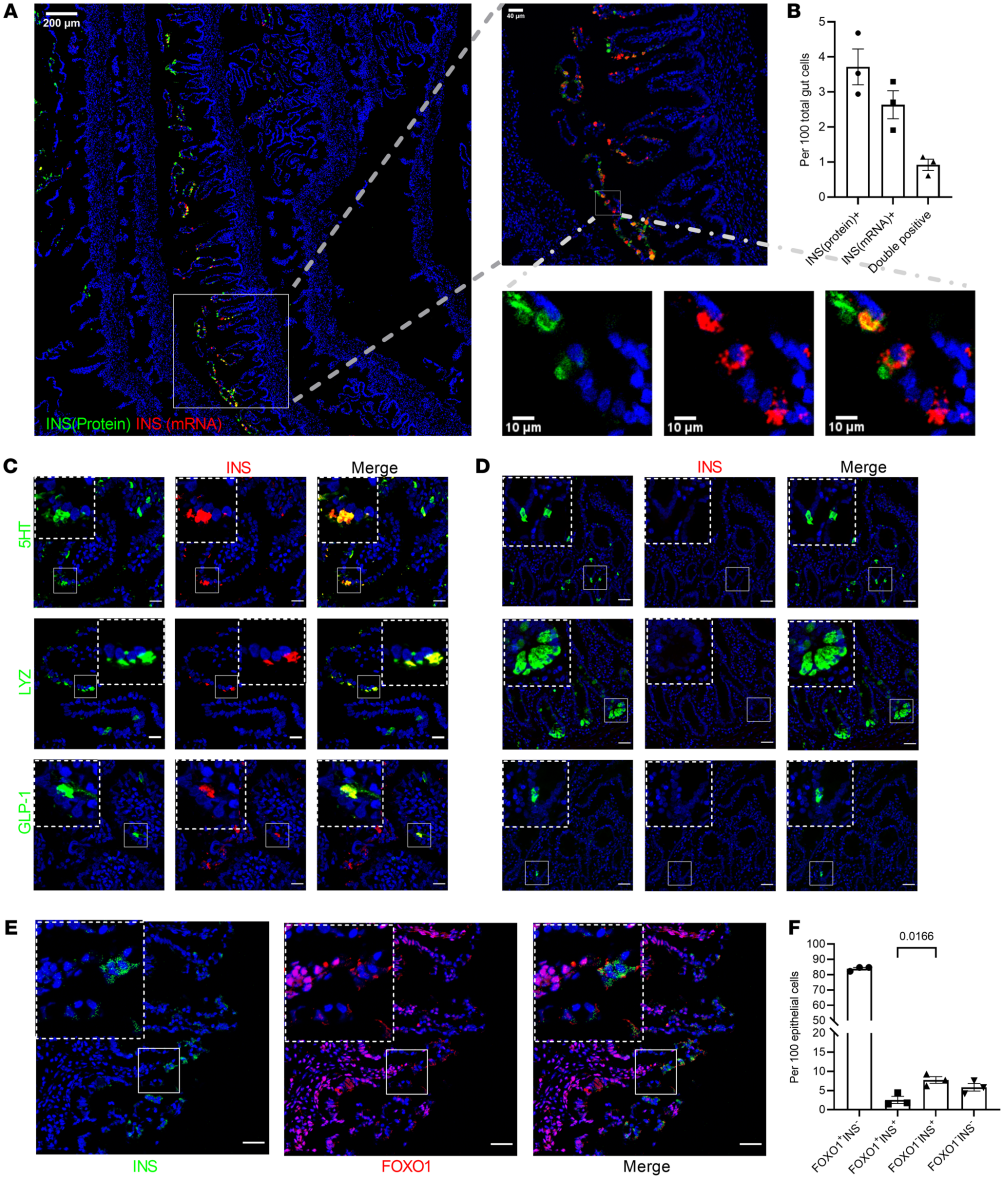
INSULIN and FOXO1 expression in human fetal small intestine secretory lineage cells. (**A**) Representative image (GA = 17 weeks) of tile scanning of one-fourth fetal proximal intestinal roll section stained with INS mRNA in red and INS protein in green. (**B**) Quantification of INS protein^+^, *INS* mRNA^+^, and double-positive cells. *n* = 3 different donors. GA = 15–17 weeks. Data are represented as mean ± SEM. (**C**) Insulin (red) and 5HT, lysozyme, or GLP-1 (green) staining in fetal human anterior intestine (GA = 17 weeks). Colocalization is shown in yellow. Scale bars: 20 μm. (**D**) Insulin (red) and 5HT, lysozyme, or GLP-1 (green) staining in adult human duodenum. Colocalization is shown in yellow. Scale bars: 40μm. (**E**) Insulin (green) and FOXO1 (red) staining in fetal human anterior intestine. Scale bars: 20 μm. (**F**) Quantification of FOXO1^–^Insulin^+^ versus FOXO1^+^Insulin^+^ cells in fetal human proximal intestine. *n* = 3 different donors. Each point shows averaged counting value from 3 to 4 different images per donor. Data are represented as mean ± SEM. Two-tailed *t* test.

Pancreas and small intestine share a common endodermal origin. Recent studies show that enteroendocrine K/L cells express insulin during fetal, but not postnatal, life (19). These data provide a plausible developmental explanation for the observation that *FoxO1* deletion in *Neurog3*^^+^^ endocrine progenitors generates gut β-like, insulin-secreting cells in a cell-autonomous manner (17), since *FoxO1* is generally activated upon terminal differentiation in a variety of cell types (22, 23). We tested the relationship between *FOXO1* expression/activity and insulin immunoreactivity in the human fetal intestine by analyzing whole rolls of small intestine from one 15-week gestational age (GA) subject, two 17-week GA subjects, and one 19-week GA subject. Combined immunohistochemistry and in situ hybridization detected cells coexpressing insulin mRNA and protein in fetal human intestine at 15 to 17 weeks GA (Figure 1, A and B), but barely in 19 weeks GA (especially in the villus tip pattern). Interestingly, only one-third of cells expressing insulin (*INS*) mRNA also expressed the insulin protein, consistent with the possibility that fetal intestinal insulin expression is transient (Figure 1B), likely restricted to the early second trimester. Immunostaining also revealed coreactivity with intestinal secretory cell-type markers 5HT (enterochromaffin), lysozyme (Paneth), and GLP-1 (EEC K/L cell) (Figure 1C). Double-positive 5HT/insulin and lysozyme/insulin cells were detected more frequently at the tip of villi in the proximal small intestine, whereas double-positive GLP-1/insulin cells were mainly located in the distal region. No insulin protein and RNA-positive cells were found in adult human intestine biopsies (Figure 1D and Supplemental Figure 1A; supplemental material available online with this article; https://doi.org/10.1172/JCI162720DS1). INS antibody specificity was tested using negative and positive control samples (Supplemental Figure 1B). Costaining with FOXO1 indicated that most insulin-positive cells did not express FOXO1, consistent with the possibility that *FoxO1* ablation in rodents or organoids recapitulates a developmental stage in human fetal intestine (Figure 1, E and F).

### Separate subsets of Neurog3 lineage yield intestinal β-like cells.

**Figure 2:**
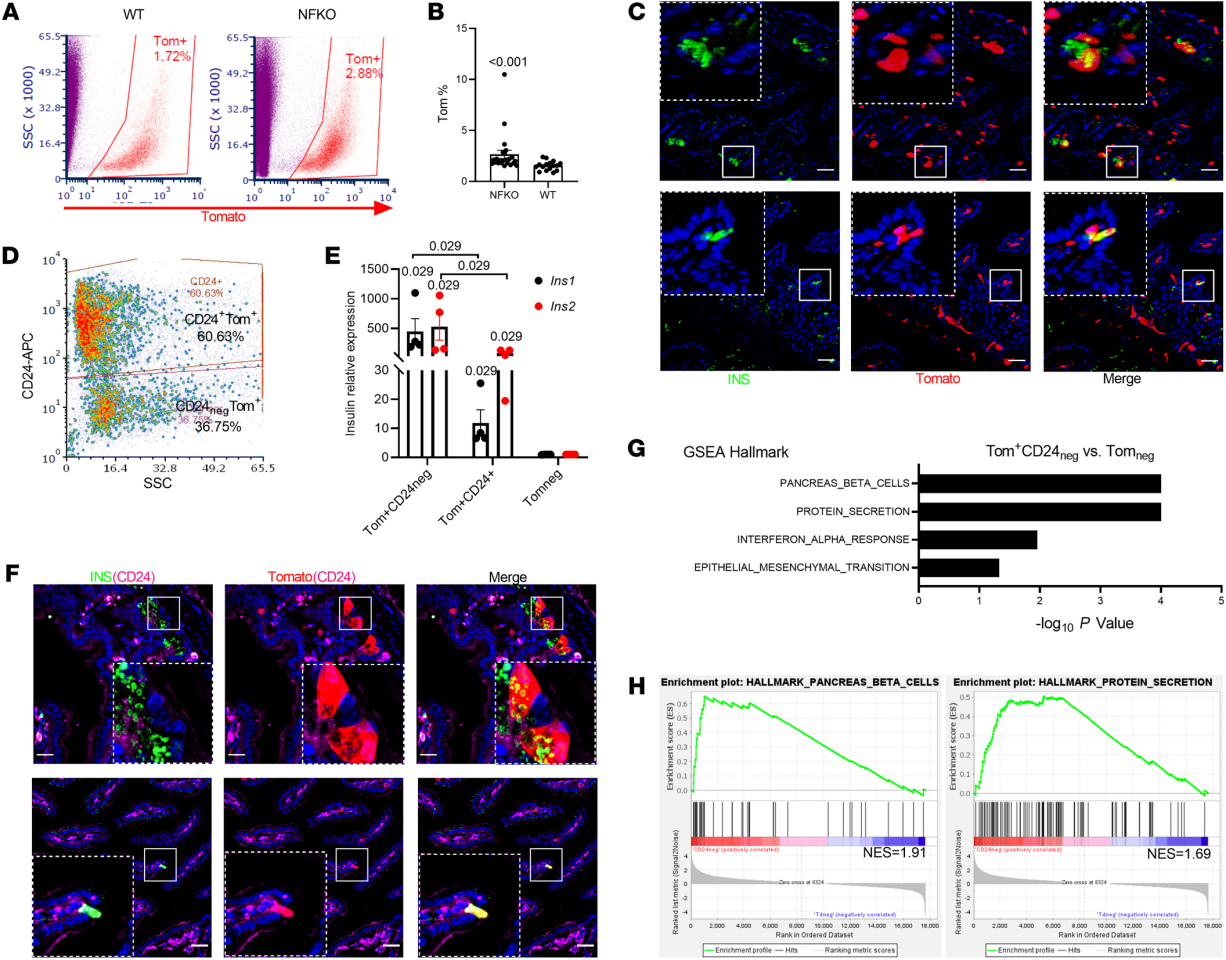
Expanded Neurog3 lineage and β-like cells in gut of Neurog3 FoxO1-KO mice. (**A**) FACS of isolated Tomato^+^ cells from either *Neurog3*Cre^+^FoxO1^fl/fl^; ROSA^tdTomato^ (NFKO) or *Neurog3*Cre^+^; ROSA^tdTomato^ (WT) gut epithelial cells. Red gate indicates sorting window for *Neurog3*-derived Tomato^+^ cells. (**B**) Tomato^+^ cell frequency assessed by FACS in NFKO and Neurog3Cre (WT) mice (NFKO, *n* = 23; WT, *n* = 16 mice). Data are represented as mean ± SEM. Two-tailed *t* test. (**C**) Representative IHC images of 2 types of Neurog3-derived β-like cells from NFKO mice: Paneth pattern (upper panel) and EEC pattern (lower panel). Scale bars: 40 μm. (**D**) FACS plot of CD24-based sorting strategy of dissociated Tomato^+^ single cells from NFKO small intestinal epithelial cells. SSC, side scatter. (**E**) *Ins1* and *Ins2* mRNA in sorted CD24^+^Tomato^+^, CD24^neg^Tomato^+^, and Tomato^neg^ populations (*n* = 4 mice, Mann-Whitney rank-sum test). (**F**) Representative IHC of insulin, CD24, and Tomato. Paneth (upper panels) and EEC pattern (lower panels) of CD24 staining in insulin^+^ cells (green and red channel double colocalization is shown in yellow; green, red, and magenta triple colocalization is shown in white). (**G** and **H**) Enriched hallmark gene sets in CD24^neg^Tomato^+^ versus Tomato^neg^ population predicted by the GSEA.

The discovery of cells with mixed insulin/Paneth/goblet features is consistent with the notion that different cell types arise from *Neurog3^+^* progenitors: EEC, goblet, and Paneth cells (Supplemental Figure 4A) (24). Therefore, we asked whether the latter two subtypes also give rise to gut β-like cells because this finding would greatly expand the repertoire of target cells for conversion to β-like cells. To answer this question, we used a 2-step enrichment procedure of in vivo lineage tracing with *Neurog3*Cre-*FoxO1^fl/fl^*; Rosa26^^tdtomato^^ mice to label *FoxO1*-KO cells derived from *Neurog3* progenitors (NFKO), followed by CD24 immunostaining to distinguish among EEC, goblet, and Paneth cells (Supplemental Figure 2) (25). *FoxO1* expression was significantly reduced in sorted *Neurog3^^+^^-*derived Tomato^^+^^ cells from NFKO mice, while *FoxO3* and *FoxO4* were unchanged (Supplemental Figure 3). Quantitative flow cytometry analysis (FACS) revealed that NFKO increased Neurog3^^+^^-derived cells approximately 1.7-fold, from 1.54% to 2.65% (*P* < 0.0001) (Figure 2, A and B). Single-cell RNA-Seq (scRNA-Seq) showed an expansion of the EEC and goblet/Paneth lineages among Neurog3 daughter cells of NFKO mice (Supplemental Figure 4, A and B). Interestingly, both subpopulations included insulin-immunoreactive cells (Figure 2C). CD24 staining allowed us to subdivide *Neurog3^^+^^-*derived (Tomato^^+^^) cells into 2 distinct populations: CD24^^neg^^Tomato^^+^^ and CD24^^+^^Tomato^^+^^ (Figure 2D). CD24^^+^^ cells included both Paneth and 5HT cells (Supplemental Figure 2). Quantitative PCR (QPCR) data demonstrated that CD24^^neg^^Tomato^^+^^ cells from NFKO mice were highly enriched in *Ins1* and *Ins2* mRNA (500- to 1,000-fold), while CD24^^+^^Tomato^^+^^ cells showed a more limited 10- to 30-fold enrichment (Figure 2E). Consistently, insulin-immunoreactive cells showed weak or absent CD24 membrane staining (Figure 2F). Notably, gene-set enrichment analysis (GSEA) of bulk RNA-Seq data showed increased pancreatic β cell– and protein secretion–related transcripts in the CD24^^neg^^Tomato^^+^^ population (Figure 2, G and H). The morphology and gene expression profiles of these cells was consistent with a dual origin from EEC and Paneth/goblet cells (Figure 2, C and F). Besides Paneth/goblet lineage markers (*Spink4*, *Defa24*, *Muc2*, *Lyz1*) and EEC lineage markers (*Chga*, *Gcg*, *Tph1*, *Cck*, *Pyy*), we detected quiescent stem cell markers *Hopx* and *Olfm4* in the insulin-immunoreactive population (Supplemental Figure 4, C and D). Moreover, CytoTRACE analysis (26) showed that the insulin-immunoreactive population represents a less differentiated cell state compared with insulin-negative cells from NFKO mice (Supplemental Figure 4E), supporting the notion that *FoxO1* ablation in *Neurog3*-derived cells brings about a fetal-like stage.

### Lineage tracing identifies a dual source of β-like cells following FoxO1 ablation.

**Figure 3:**
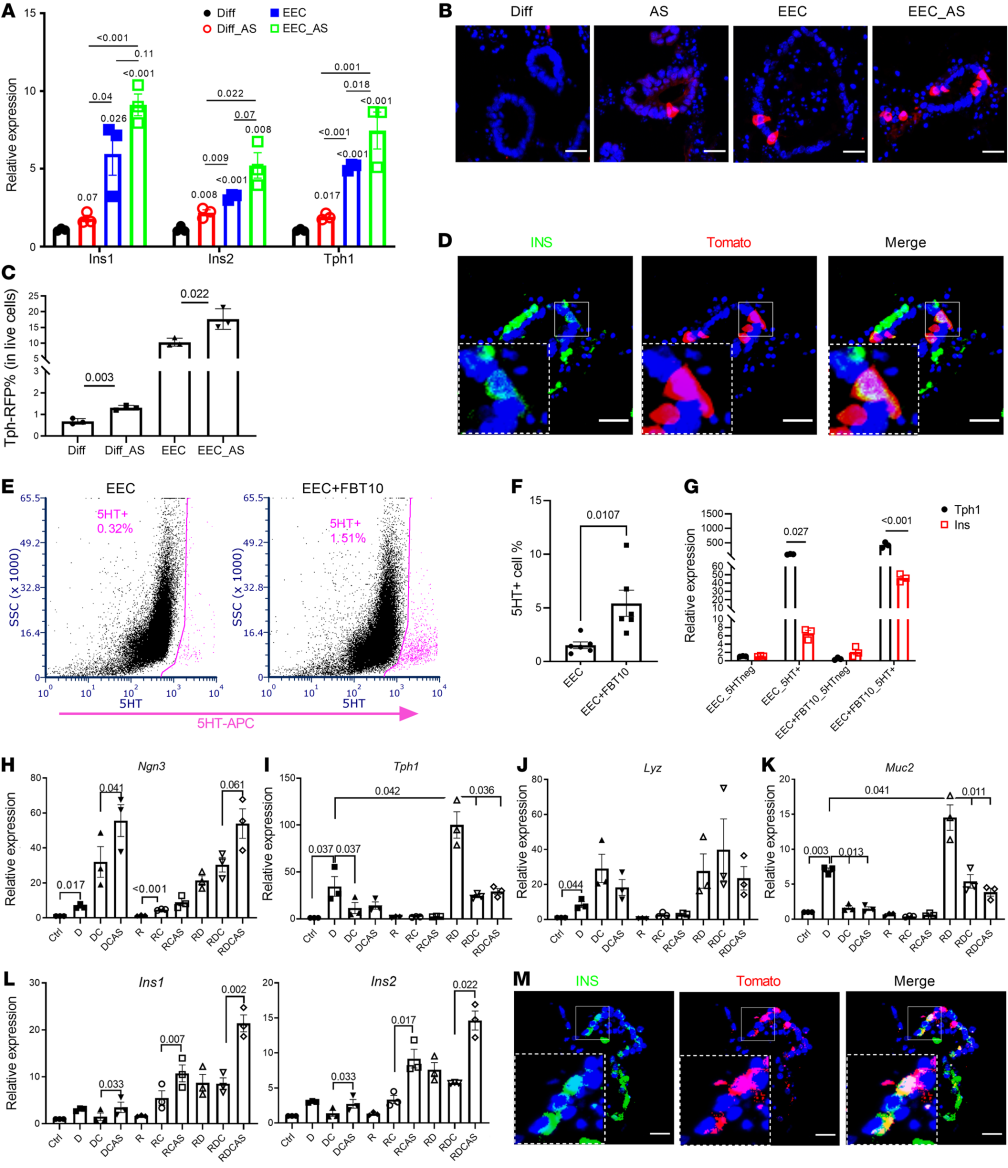
Dual source of β-like cells in murine and hGOs. (**A**) QPCR of mouse intestinal organoids following EEC induction. *n* = 3 independent experiments. Data are represented as mean ± SEM. Paired *t* test. (**B**) Tomato staining of *Tph1*Cre^ERT2^ organoids after 4 days in differentiation (Diff) and EEC induction medium following activation of Tomato reporter. *n* = 3 independent experiments. Scale bars: 20 μm. (**C**) Percentage of *Tph1*Cre^ERT2^ Tomato cells in mouse GOs with or without iFOXO1 (AS) treatment (*n* = 3 independent experiments). (**D**) Lineage tracing of *Tph1*Cre^ERT2^ 4 days after activation of Tomato expression. Scale bars: 20 μm. (**E**) FACS diagram representing the sorted 5HT^+^ (pink) population in EEC induced from hGOs with or without FBT10 treatment. (**F**) Percentage of 5HT^+^ cells in EEC induced from hGOs with or without FBT10 treatment. *n* = 6 independent experiments. Data are represented as mean ± SEM. Two-tailed *t* test. (**G**) QPCR of *Ins* and *Tph1* in sorted 5HT^+^ cells with or without FBT10 treatment. *n* = 3 independent experiments. (**H**–**L**) QPCR of different marker genes following treatment with combination of iNotch (DAPT [D]), iTGF-β (Repsox [R]), iGSK3β (Chir [C]); and iFOXO1 (AS). *n* = 3. Data are represented as mean ± SEM. Paired *t* test. (**M**) Lineage tracing of *Lyz1*Cre^ER^ 4 days after activation of Tomato expression. Scale bars: 20 μm.

The presence of 2 distinct cell subtypes with β-like features raised the possibility that other intestinal cell types can be converted to insulin-immunoreactive cells by *FoxO1* ablation. To critically test this hypothesis, we assessed generation of β-like cells by tracing the 5HT lineage using primary organoids from *Tph1*Cre^^Ert2/+^^; Rosa26^^tdTomato^^ mice to identify EEC-derived β-like cells, and the goblet/Paneth lineage using *Lyz1*Cre^^Er/+^^; Rosa26^^tdTomato^^ organoids. After inducing *Tph1* reporter-dependent gene activation with 4-OH-TAM, we enriched organoids in EEC by incubating them in medium containing inhibitors of Notch, WNT, and MEK (DAPT, IWP2, and PD0325901, respectively) (27). To induce conversion into β-like cells, we added the chemical FOXO1 inhibitor AS1842856 (AS) (28, 29). QPCR analysis showed significant increases of *Ins1*, *Ins2*, and *Tph1* mRNA after incubation in EEC medium with or without AS (Figure 3A). We found approximately 11% 5HT cells by immunostaining and flow cytometry (Figure 3, B and C). Treatment with AS increased the percentage of 5HT cells 1.5-fold (Figure 3C). Pulse-chase labeling also showed that after 4-OH-TAM treatment, insulin-positive cells colocalized with newly generated 5HT cells (Tomato^^+^^ cells) regardless of whether organoids had been subjected to the EEC differentiation protocol (Figure 3D and Supplemental Figure 5). Induction of 5HT-positive cells was also evident in EEC-enriched human GOs (hGOs) (Figure 3, E and F). In sorted 5HT-positive cells from hGOs, insulin mRNA was induced approximately 6-fold by incubation in EEC medium and 30-fold by addition of another FOXO1 inhibitor, FBT10 (Figure 3G) (30, 31). These data are consistent with the hypothesis that FOXO1 inhibition facilitates conversion of 5HT cells into β-like cells.

Next, we performed similar experiments in organoids derived from *Lyz1*Cre^^Er/+^^; Rosa26^^tdTomato^^ mice to label goblet/Paneth cells, followed by induction of these 2 interrelated lineages (Figure 3H). We optimized chemical induction of the Paneth/goblet lineage by different combinations of the glycogen synthase kinase-3β (GSK3β) inhibitor Chir99021, Notch inhibitor DAPT, and TGF-β inhibitor Repsox (32, 33). QPCR data showed that single Notch inhibition enriched all secretory cell markers, such as *Neurog3*, *Tph1*, *Lyz1*, and *Muc2*; this effect was strengthened by the TGF-β inhibitor. In contrast, addition of the GSK3β inhibitor increased *Neurog3* and *Lyz1*, but decreased *Tph1* and *Muc2*, consistent with the possibility that this combination promotes an earlier stage of EEC differentiation. Single treatment with either TGF-β or GSK3β inhibitor had no effect (Figure 3, H–K). The triple combination of Notch, TGF-β, and GSK3β inhibitors resulted in a 10-fold induction of *Ins1* and *Ins2* mRNA along with EEC progenitor markers, indicating a trend toward the β cell–like phenotype. The effect of the triple blockade was amplified by adding the FOXO1 inhibitor AS, with both *Ins1* and *Ins2* mRNA expression increasing by 20- and 14-fold, respectively (Figure 3L). Adding AS also strengthened the effect of the dual Notch/TGF-β or Notch/GSK3β blockade (Figure 3L). To confirm the origin of β-like cells, we performed immunohistochemistry and found that insulin colocalized with LYZ1-tomato cells, indicating that β-like cells can also arise from Paneth/goblet cells (Figure 3M).

### Newly developed FOXO1 inhibitors increase β-like cell conversion.

**Figure 4:**
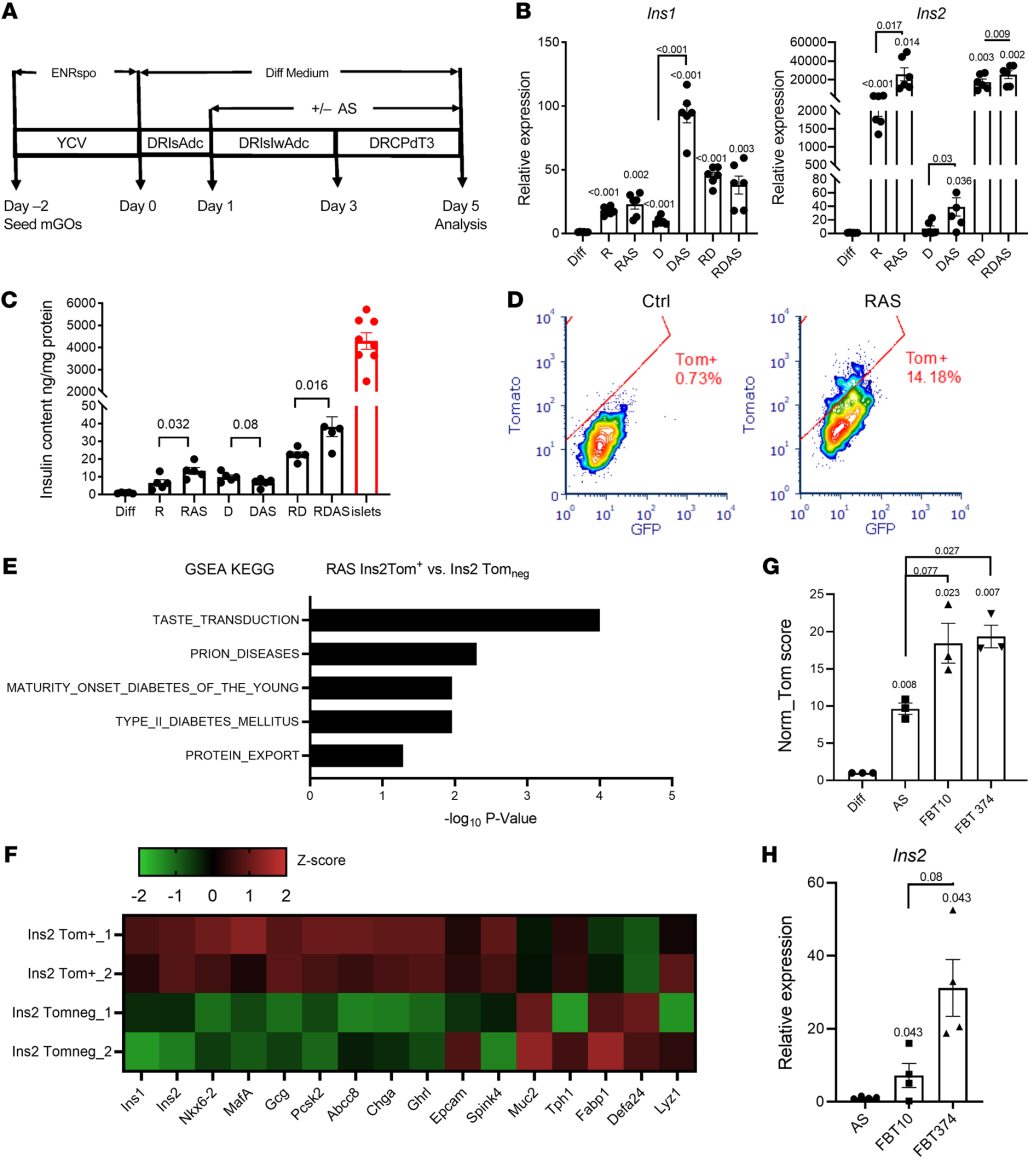
Conversion of gut cells into insulin-producing cells by combination treatment. (**A**) Modified differentiation protocol to induce conversion of INS^+^ cells by the addition of FOXO1 inhibitor to the differentiation medium at different stages. Treatment details are explained in *Quantitative measurement of conversion insulin-producing cells with cultured organoids* in Supplemental Methods. ENRspo, organoid growth medium epidermal growth factor/Noggin/R-spondin; Y, Y-27632; C, CHIR99021; V, vaproic acid; D, DAPT; R, Repsox; Is, ISX-9; Adc, 5-Aza-2′-deoxycytidine; Iw, IWP2; Pd, PD0325901; T3, thyroid hormone. (**B**) QPCR of *Ins1* and *Ins2* expression from organoids following treatment with the differentiation cocktail, comprising inhibitors of Notch (DAPT [D]) and/or TGF-β (Repsox [R]), followed by the addition of FOXO1 inhibitor (AS). *n* = 6 independent experiments. Data are represented as mean ± SEM. Paired *t* test. (**C**) Insulin content in organoids following treatment with the differentiation cocktail compared with islets. *n* = 4 independent experiments. Data are represented as mean ± SEM. One-way ANOVA. (**D**) FACS diagram presenting the percentage of converted INS2^+^ cells in control versus differentiation cocktail-treated organoids. (**E**) Upregulated KEGG pathways in sorted INS2-Tom^+^ cells versus INS2-Tom^neg^ cells. (**F**) Heatmap comparing expression levels of typical islet and gut epithelial marker genes in sorted INS2-Tom^+^ versus INS2-Tom^neg^ from differentiated organoids of INS2-Tomato mice. (**G**) Comparative evaluation of the potencies of 2 new FBT compounds and iFOXO1 (AS) to generate insulin^+^ cells by an integrated calculation (Tom score) of INS2-Tomato intensity, percentage of INS2-Tomato cells, and live-cell percentage, as detected by flow cytometry. *n* = 3 independent experiments. Data are represented as mean ± SEM. Paired *t* test. (**H**) *Ins2* relative expression in sorted INS2-Tomato cells. *n* = 4 independent experiments. Data are represented as mean ± SEM. Paired *t* test.

Based on these data, we sought to increase the efficiency of generating β-like cells using a modified secretory cell conversion protocol combined with FOXO1 inhibition in mouse intestinal organoids (34) (Figure 4A). TGF-β inhibition resulted in strong induction of *Ins1* and *Ins2*; the latter was further increased by approximately 2-fold by the FOXO1 inhibitor AS. Notch inhibition had a stronger effect on *Ins1* than on *Ins2*, and addition of AS increased both. Addition of the Notch inhibitor to the TGF-β blockade, either in the presence or absence of AS, had no effect, indicating that Notch is epistatic to TGF-β in the pathway leading to β-like cell conversion (Figure 4B and Supplemental Figure 6). Measurements of insulin content in organoid extracts are consistent with the mRNA findings and show that FOXO1 inhibition increased the amount of insulin recovered after single TGF-β or dual Notch/TGF-β blockade (Figure 4C).

Next, we set up quantitative assays with cultured GOs derived from mice bearing a RIP-Cre; Rosa26^^tdtomato^^ reporter allele to evaluate the efficiency of β-like cell conversion. In this assay, we used FACS to separate and quantitate cells in which *Ins2* expression had been activated, as indicated by the Tomato reporter, from non–insulin-expressing epithelial cells. In a typical experiment, about 14% of cultured organoid cells demonstrated onset of red fluorescence following combined TGF-β/FOXO1 inhibition (Figure 4D). Using this screening platform, we validated several FOXO1 inhibitors (FBT) based on their potency in reporter promoter assays (20). We selected 2 compounds, FBT10 and FBT374, that outperformed AS in conversion frequency to β-like cells and *Ins2* expression (Figure 4, G and H). Using Tomato^^+^^ (β-like) cells isolated by FACS, we compared expression of β cell–specific genes between converted INS2-Tom^^+^^ β-like cells versus INS2-Tom^^neg^^ cells. RNA-Seq showed that organoid-derived insulin-immunoreactive cells expressed pancreatic β cell markers, including *Nkx6.2*, *MafA*, *Pcsk2*, and *Abcc8* (Figure 4F), while also retaining some intestinal epithelial markers (Supplemental Figure 7). GSEA of the KEGG pathway revealed that Ins2-Tom^^+^^ cells were highly enriched in genes related to biological processes in maturity-onset diabetes of the young and type 2 diabetes (Figure 4E). Thus, intestinal β-like cells obtained by FOXO1 inhibition share a common molecular signature with islet β cells.

### Inhibition of Notch and TGF-β in FoxO1-deficient mice increases Neurog3^^+^^ and β-like cells.

**Figure 5:**
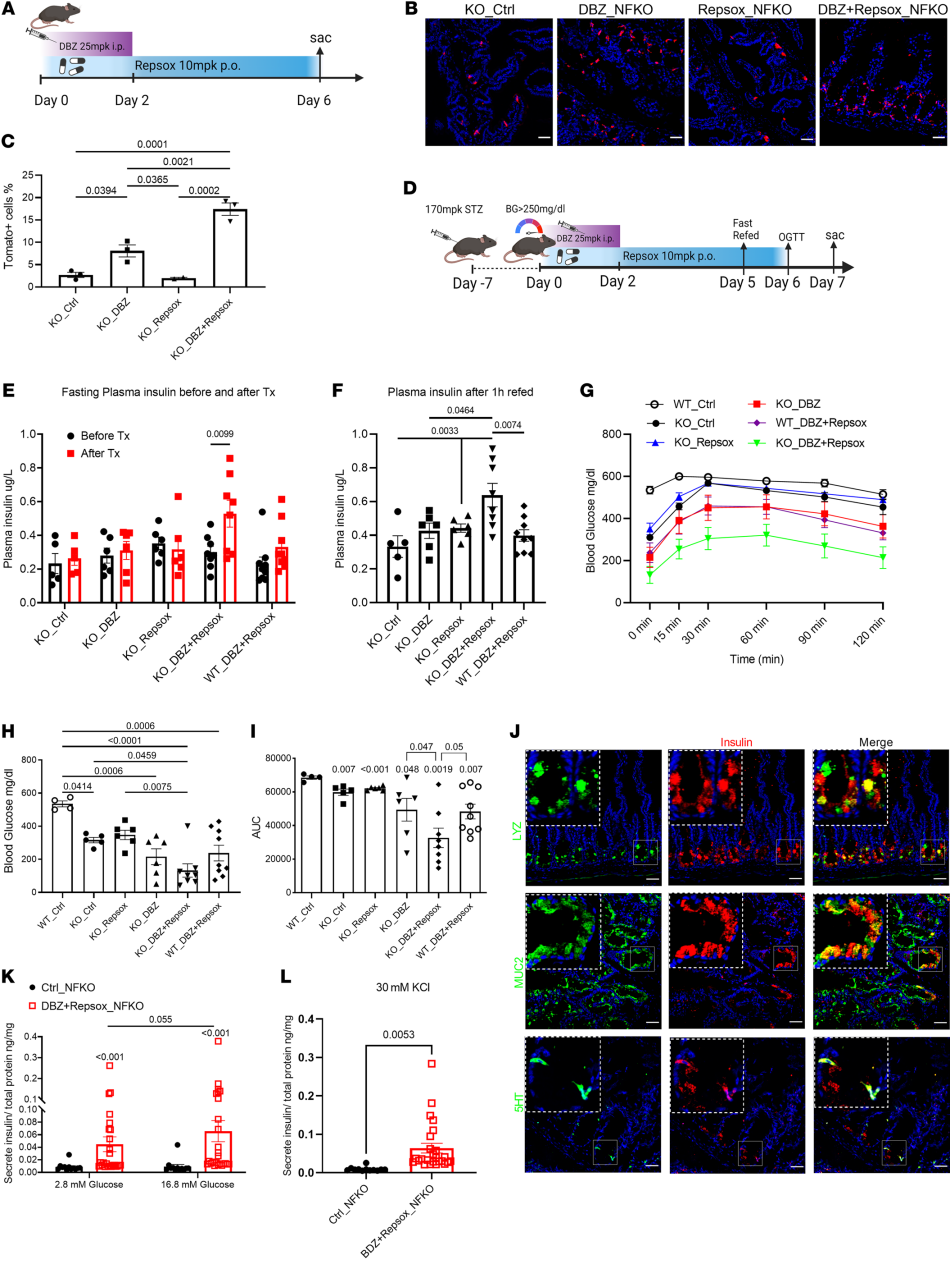
iNotch and iTGF-β combination therapy generates insulin^+^ cells in NFKO mice. (**A**) Experimental design for DBZ and Repsox combination treatment of NFKO mice. (**B**) Representative IHC images of Tomato (red) staining in *Neurog3*Cre *FoxO1^fl/fl^*; ROSA tdTomato mice following DBZ, Repsox, or combination treatment. Scale bars: 40 μM. (**C**) Tomato^+^ cell frequency measured by FACS in control, DBZ-, Repsox-, or combination-treated *Neurog3*Cre *FoxO1^fl/fl^*; ROSA tdTomato mice. *n* = 3 mice in each treatment group. Data are represented as mean ± SEM. Two-way ANOVA. (**D**) Experimental design for DBZ and Repsox combination treatment in STZ-WT or NFKO mice. (**E**) Four-hour fasting plasma insulin levels in STZ-NFKO and STZ-WT mice before and after treatment with the indicated compounds. (**F**) One-hour refed plasma insulin levels in STZ-NFKO and STZ-WT mice before and after treatment with the indicated compounds. (**G**) OGTTs after DBZ, Repsox, and dual treatment. (**H**) Four-hour fasting glucose level measurements before OGTT. (**I**) AUC of OGTT shown in **G**. (**J**) Representative IHC images of lysozyme (upper panels, green), MUC2 (middle panels, green), and 5HT (lower panels, green) costained with insulin (red) in combination therapy–treated STZ-NFKO mice. Scale bars: 40μm. Green and red channel colocalization shown in yellow. *n* = 6 to 9 mice in each treatment group. Data are represented as mean ± SEM. Two-way ANOVA. (**K** and **L**) Glucose or KCl stimulates insulin secretion from duodenum of control or DBZ and Repsox combination–treated NFKO mice. *n* = 12–25. Data are represented as mean ± SEM. Mann-Whitney rank-sum test.

Next, we tested to determine whether triple blockade of Notch, TGF-β, and FOXO1 can induce cell conversion in vivo. To this end, we combined genetic *FoxO1* ablation in *Neurog3*cre-*FoxO1^fl/fl^*; Rosa26^^tdtomato^^ mice with pharmacological treatment with Repsox and a different Notch inhibitor, the γ-secretase inhibitor DBZ. We injected DBZ for the first 2 days, followed by 5 days of Repsox oral dosing (21, 35) (Figure 5A). Immunohistochemistry and quantitative FACS showed that DBZ increased *Neurog3*-derived cells by approximately 2.5-fold and the DBZ/Repsox combination by 7-fold, to account for approximately 15% of all live duodenal epithelial cells. Repsox had no effect by itself (Figure 5, B and C, and Supplemental Figure 8A). The treatment had similar effects on the percentage of 5HT cells, consistent with data in cultured organoids (Supplemental Figure 8, B and C). These data show that triple inhibition expanded the EEC lineage, a necessary condition for β-like conversion of a subset of cells.

To determine whether these treatments resulted in the formation of functional gut β-like cells, we rendered the animals diabetic with streptozotocin (STZ) and measured the effects of the various interventions on fasting glucose, glucose tolerance, plasma insulin, and generation of insulin-immunoreactive intestinal cells (Figure 5D). *FoxO1* ablation resulted in lower fasting glycemia and improved glucose tolerance tests (GTTs) after STZ administration, consistent with prior observations (17). After a 6-day course of dual inhibition with Repsox and DBZ in *FoxO1*-KO mice, plasma insulin levels increased following a 4-hour fast or 1-hour refeeding (Figure 5, E and F). In oral GTTs (OGTTs), this treatment yielded the largest improvement compared with vehicle or any single treatment or dual Repsox and DBZ inhibition in WT controls (Figure 5, G–I). Dual inhibition increased 5HT and goblet cell population while decreasing the Paneth cell marker lysozyme (Supplemental Figure 9). Immunohistochemistry revealed abundant insulin-immunoreactive cells within intestinal crypts and colocalization with 5HT and lysozyme/MUC2 (Figure 5J) as well as pancreatic β cell markers PC2, MAFA, and SUR1 (Supplemental Figure 12), suggesting that these cells undergo conversion to β-like cells. Moreover, the various treatments had no effects on residual endocrine pancreas β cells (Supplemental Figure 10, A and B), total pancreatic insulin content (Supplemental Figure 10C), and proliferating β cells, as assessed by KI-67 staining (Supplemental Figure 10D). DBZ alone or combined with Repsox similarly enhanced circulating GLP1 levels (Supplemental Figure 11, A and B) as well as the number of GLP1- and GIP-expressing cells in the small intestine (Supplemental Figure 11, C–F). As these treatments had no effect on plasma insulin and pancreatic insulin content was negligible anyway, the increased plasma insulin and lower glycemia seen in Repsox/DBZ-treated *FoxO1*-KO mice should be attributed to the induction of intestinal insulin-positive cells rather than expansion of other EEC lineages. Furthermore, we performed glucose- and KCl-induced insulin secretion assays from gut isolated from Repsox/DBZ-treated *FoxO1* KOs and found that these intestinal β-like cells release insulin in response to secretagogues (Figure 5, K and L). Taken together, these data indicate that combined FOXO1, Notch, and TGF-β inhibition increases the efficiency of cell conversion in vivo and is associated with a commensurate glucose-lowering effect in diabetic animals.

### Triple combination therapy lowers blood glucose and induces gut β-like cells in NOD mice.

**Figure 6:**
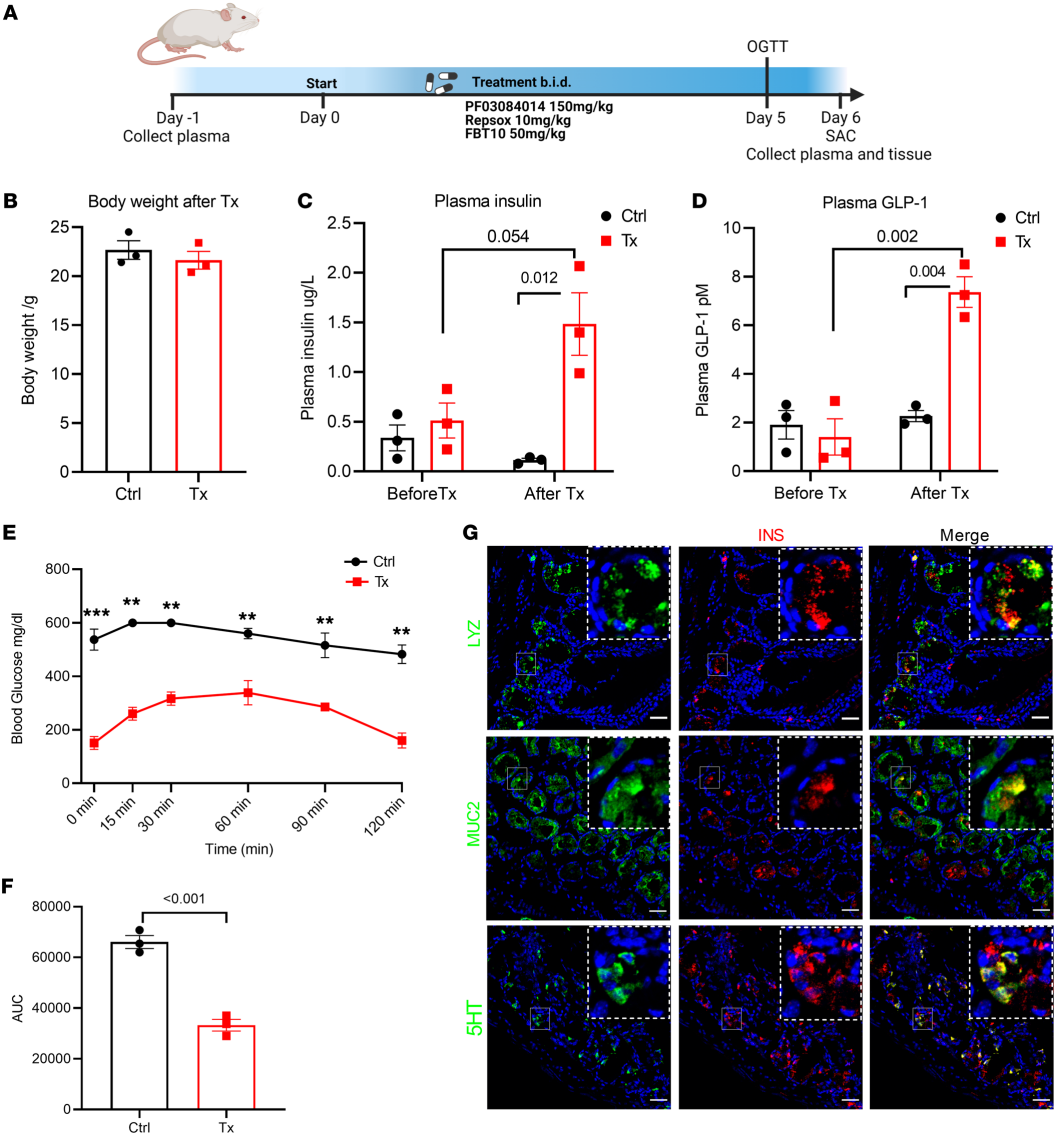
Triple combination therapy lowers blood glucose and induces gut β-like cells in NOD mice. (**A**) Experimental design for PF-03084014, Repsox, and FBT10 triple combination treatment of NOD mice.(**A**) Body weight measurement after 5 days of combination treatment in control (vehicle) and Tx (treatment) groups. *n* = 3 mice each group. Data are represented as mean ± SEM. Two-tailed *t* test. (**C** and **D**) Plasma insulin and GLP-1 in control and treatment groups before and after 5-day treatment. (**E**) OGTTs after 5 days of vehicle or triple combination treatment. (**F**) AUC of OGTT shown in **E**. (**G**) Representative IHC of lysozyme (upper panels, green), MUC2 (middle panels, green), and 5HT (lower panels, green) costained with insulin (red) in triple combination therapy–treated NOD mice; green and red channel colocalization shown in yellow. Scale bars: 40 μm. *n* = 3 mice each group. Data are represented as mean ± SEM. Two-tailed *t* test.

To evaluate the translational value of this triple combination therapy in an autoimmune model of diabetes, we used Repsox and the γ-secretase inhibitor PF-03084014, currently in phase II trials for the treatment of different forms of cancer (36), in combination with the chemical FOXO1 inhibitor FBT10 (Figure 6A) (20, 21). Five days of oral administration with FBT10, PF, and Repsox only slightly decreased body weight (Figure 6B), with a significant increase of plasma insulin and GLP1 levels (Figure 6, C and D). Triple combination therapy decreased 4-hour fasting blood glucose levels by 400 mg/dl and nearly normalized OGTT compared with that of vehicle-treated controls (Figure 6, E and F). Immunohistochemistry confirmed the presence of β-like cells in the intestine of the treatment group, partly coimmunoreactive with 5HT or lysozyme/MUC2, but not in the vehicle group (Figure 6G and Supplemental Figure 13A). There were no differences in residual islet β cells, and both groups showed evidence of islet immune cell infiltration (Supplemental Figure 13B).

### Combination treatment of hGOs induces insulin^^+^^ cells.

**Figure 7:**
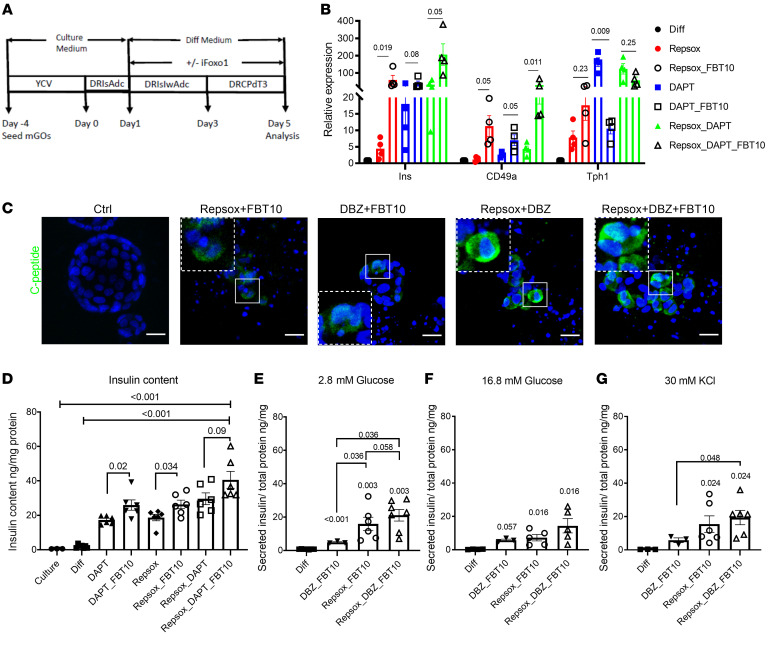
Induction of β-like cells by combination treatment in hGOs. (**A**) Schematic showing treatment protocol. (**B**) QPCR of different marker genes from hGOs treated with the differentiation cocktail. Data are represented as mean ± SEM. Paired *t* test. (**C**) Representative IHC images of C-peptide (green) staining in hGOs treated with differentiation cocktail. Scale bars: 20 μm. (**D**) Insulin content of the differentiation cocktail–treated hGOs. *n* = 6 independent experiments. Data are represented as mean ± SEM. One-way ANOVA. (**E**–**G**) Glucose and KCl stimulate insulin secretion from hGOs treated with different cocktails. *n* = 3 independent experiments, 1–3 replicates in each experiment. Data are represented as mean ± SEM. Two-tailed *t* test.

Finally, we determined the effects of triple chemical blockade of Notch, TGF-β, and FOXO1 using primary human duodenal organoids (Figure 7A). QPCR analysis indicated a remarkable induction of insulin and CD49a, a membrane marker of hESC-derived β cells, by the triple combination treatment (7) (Figure 7B). Measurements of insulin content and C-peptide immunohistochemistry confirmed these findings (Figure 7, C and D). The β-like cells generated from hGOs showed insulin secretion. Interestingly, similarly to ES-derived β-like cells, they failed to clearly respond to high glucose or KCl (Figure 7, E–G). This likely reflects an immature stage due to the short course of the differentiation experiment.

## Discussion

Pancreas and intestine share a common developmental origin, and their endocrine compartments share a common progenitor as well as several terminally differentiated cell types, such as somatostatin- (SST-) and ghrelin-producing cells. Other cells, for example α- and K/L cells, give rise to alternatively spliced products of the same preproglucagon gene in the 2 organs (37). Insulin-producing cells are, however, restricted to the pancreas. We were therefore quite surprised when, a decade ago, we observed that genetic ablation of *FoxO1* in endocrine progenitors resulted in the generation of intestinal cells with highly differentiated β-like cell features normally only found in pancreatic islets. Three subsequent pieces of evidence clarified this finding. First, Stanger and Zhou independently replicated these findings using a forced expression approach with NEUROG3, PDX1, and MAFA (15, 16), confirming the potential of the gut to undergo this conversion. Next, we showed that FOXO1 inhibition appeared to reprogram enterochromaffin 5HT cells into β-like cells in hGOs (18). And the Melton laboratory reported that enterochromaffin-like cells are a “byproduct” of stem cell differentiation into β cells, including expression of genes related to 5HT biosynthesis. The similarities between these 2 cell populations suggest that there is a relationship between enterochromaffin and β cell fates (7). This, in turn, dovetails with the notion that pancreatic β cells synthesize 5HT (38). Finally, the recent description of bona fide insulin-producing cells in the fetal human gut suggests that FOXO1 ablation is arresting Neurog3 progenitor differentiation at a fetal-like stage (19), providing a plausible underpinning as well as unifying mechanism for these disparate observations.

One unexplained feature of these findings was that other secretory lineage cells also arise from *Neurog3* progenitors (24). In this regard, it is noteworthy that the noncanonical WNT/planar cell polarity pathway, which controls islet β cell functional heterogeneity, primes intestinal stem cells toward the EEC or Paneth lineages (39). In this work, we provide direct lineage-tracing evidence that secretory cells in the nonendocrine (Paneth and goblet) fate can also be converted to intestinal β-like cells. In addition, expanding on a recent communication (19), we show that in human fetal intestine of 15- to 17-week GA, insulin-immunoreactive cells also colocalize with goblet/Paneth lineage markers, but exclude active FOXO1, lending further support to the notion that FOXO1-inactive cells can be converted to β-like cells. These findings address the question of which type of cell can be converted into insulin-immunoreactive β-like cells, extending previous observations (17, 18, 21).

The data above raised the possibility that leveraging additional signaling pathways can modulate the conversion process in synergy with FOXO1. TGF-β, WNT, FGF, Notch, BMP, and FOXO1, along with relevant receptors and signaling pathways, are involved in pancreatic and intestinal tissue patterning (40, 41). FOXO1 and Notch signaling interact in determining intestinal stem cell differentiation into Paneth/goblet (42) and EEC lineages (19, 21, 43). Thus, we combined genetic *FoxO1* KO with pharmacological Notch inhibition (DBZ) to show that dual Notch/FOXO1 inhibition expands the *Neurog3^+^* progenitor pool and its secretory lineage-cell descendants. Moreover, adding the TGF-β inhibitor Repsox further increased the *Neurog3^+^* lineage, indicating a synergistic effect on endocrine induction, as observed during the derivation of β-like cells from stem cells (4). Interestingly, TGF-β inhibition in the combination treatment decreased expression of the Paneth cell marker lysozyme, but increased the EC marker 5HT and the goblet marker MUC2, indicating that the 2 pathways affect sublineage specification. Regardless of the differentiation protocol applied, insulin expression levels in reprogrammed GO remained lower than in pancreatic islets. A similar limitation occurs when β-like cells are derived from hESCs.

Numerous studies tracking the fate of Paneth, goblet, and tuft cells and EECs have shown that lineage-committed cells are capable of dedifferentiating into multipotent ISCs during gut regeneration (44–46). Dedifferentiated cells can adopt an alternate cell fate upon injury or perturbation of the intestinal epithelium. The molecular mechanisms driving cellular reprogramming remain to be elucidated. Our findings strengthen the notion that FOXO1 participates in intestinal secretory lineage transdifferentiation, which is similar to its role in pancreatic β cells (47, 48). scRNA-Seq of *FoxO1*-ablated cells also shows the reemergence of *Hoxp*- and *Olfm4*-positive cells along with β-like cells, suggesting that committed secretory cells can revert to a stem- or fetal-like stage as a path to differentiating into β-like cells. This process too bears similarities with the role of *FoxO1* in pancreatic islets (17).

Combination treatment had a glucose-lowering effect in mice, adding to an emerging body of evidence that pharmacological FOXO1 inhibition is a viable option for β cell replacement. Although our focus was to probe the mechanistic underpinning of the conversion, the potential use of combination treatment as an alternative to insulin injections or cell transplant should be considered. Most modern treatments leverage detailed knowledge of signaling pathways to target disease processes as diverse as different types of cancer or immune disorders with combination approaches.

In summary, we characterized insulin-secreting β-like cells using genetic and pharmacologic models of signaling perturbations. Based on this insight, we developed a robust combination treatment to generate β-like gut cells in mice and cultured human enteroids. The discovery of similar cells in the human fetal intestine (19) raises the question of whether these manipulations restore a fetal-like cell type. In addition to providing developmental and mechanistic insight into this process, our findings expand potential therapeutic options for insulin replacement.

## Methods

Please refer to Supplemental Methods for comprehensive details.

### Animal studies.

Mouse strain information is shown in Supplemental Table 1. A single high-dose injection of STZ (170 mg/kg, MilliporeSigma) was administrated intraperitoneally to induce diabetes in 6- to 8-week-old male NFKO and littermate male *FoxO1^fl/fl^* (WT) mice. Mice that were not hyperglycemic within 1 week were excluded from further study. Blood glucose of 11- to 12-week-old NOD female mice was monitored at least twice per week. Treatment began immediately after blood glucose level was consistently above 250 mg/dl.

For in vivo drug treatment, STZ mice were injected intraperitoneally with 25 mg/kg DBZ q.d. for 2 days and/or gavaged with 10 mg/kg Repsox q.d. for 5 to 7 days. For NOD mice in vivo drug treatment, FBT10, PF-03084014, and Repsox were dosed orally twice daily at 50 mg/kg/dose, 150 mg/kg/dose, and 10 mg/kg/dose, respectively. In the fasting-refeeding study, mice were fasted for 4 hours followed by 1 hour refeed. In OGTT, mice were fasted for 4 hours followed by gavaging of 2 g/kg of d-glucose (MilliporeSigma). Blood glucose was measured at 0, 15, 30, 60, and 120 minutes. Blood was collected from tail veins with DPP4 inhibitor, and plasma insulin or GLP-1 was measured using the Insulin ELISA Kit (Mercodia) or the GLP-1 ELISA Kit (Crystal Chem).

### Human tissues.

Intestinal tissues or endoscopic biopsy was obtained from 8 patients from the Vanderbilt Clinic of Columbia University Irving Medical Center/Presbyterian Hospital, New York, New York, USA. All samples were deidentified, and the only clinical information collected was GA and additional fetal diagnoses. Intestinal samples ranging in age from 15 to 19 weeks of gestation were received immediately after elective terminations and fixed in 4% paraformaldehyde, dehydrated with 30% sucrose, and processed for OCT embedding, followed by sectioning and immunostaining.

### Chemicals.

All small molecule information for intestinal treatment is listed in Supplemental Table 2. DBZ was from Apexbio Technology; RepSox and PF-03084014 were from Selleck Chemical and FBT10 from ForkheadBio Therapeutics. For STZ mice in vivo treatment, DBZ and Repsox were formulated in 1% DMSO, 0.5% methylcellulose, and 0.2% Tween-80 PBS solution, respectively. For NOD mice in vivo treatment, FBT10, PF-03084014, and Repsox were formulated together into *N*,*N*-dimethylacetamide/solutol HS 15/water = 5:10:85 (v/v/v) solution, pH4–5.

### GO cultures.

For mouse small intestinal organoids, crypts were isolated using EDTA chelation from the duodenum and cultured as described (49).For human small intestinal organoids, crypts were isolated using EDTA chelation from the duodenum as previously described (50). IntestiCult Organoid Growth Medium (human) or IntestiCult Organoid Differentiation Medium (human) were used for culture or differentiation of hGOs (STEMCELL Technologies). Organoids were used prior to passage 3 for optimal efficiency of EEC and β-like cell induction.

### Intestinal epithelial cell isolation and sorting.

Single intestinal cell preparations were isolated from 4- to 6-week-old NFKO mice as described (51). Attached pancreata were removed under a dissection microscope to avoid pancreatic β cell contamination. Isolated intestinal epithelial cells were stained for 20 minutes with APC-conjugated anti-CD24 antibody and FITC-conjugated anti-Epcam antibody (BioLegend) prior to sorting using BD Influx.

### Flow cytometric analysis of epithelial cells.

Single-cell suspension was obtained by enzymatic digestion of intestinal mucosa or cultured organoids (51, 52). Suspended cells were first stained with LIVE/DEAD Cell Staining Kit (Invitrogen), then fixed in BD Cytofix fixation buffer. Cells were washed in permeabilization buffer, which was followed by intracellular staining before sorting or FACS analysis. When sorted cells were used for RNA isolation, 0.2% RNaseOUT (Invitrogen) was added to the antibody incubation and FACS buffer.

### RNA isolation and QPCR.

Organoids or sorted cells were lysed in 1 ml TRIzol (Thermo Fisher). RNA was isolated using the RNeasy Mini Kit or RNeasy Micro Kit (QIAGEN) followed by reverse transcription. RNA isolation from intercellular stained cell samples was as described (53). QPCR was performed with GoTaq qPCR Master Mix (Promega). Gene expression levels were normalized to *Hprt* using the 2^^–ΔΔCt^^ method and are presented as relative transcript levels.

### Quantitative measurement of conversion insulin-producing cells with cultured organoids.

Primary gut crypts that derived from a mouse bearing an RIP-Cre; Rosa26^^tdTomato^^ reporter allele were placed in culture and then induced to undergo cell conversion by applying a protocol based on published patent US20170349884A1 (34). Thereafter, *Ins2-*expressing cells were analyzed by flow cytometry and collected for further RNA analysis.

### In situ hybridization by RNAscope.

RNAscope was performed using the RNAscope 2.5 HD Detection Reagent RE Kit (ACD) combined with immunofluorescence according to the manufacturer’s instructions. A human insulin probe (ACD, 313571) was used to detected insulin mRNA.

### Immunohistochemistry.

Swiss rolls of small intestines were prepared from 6- to 8-week-old vehicle- or drug-treated mice and fixed in 4% PFA for 2 hours, followed by dehydration in 30% sucrose in PBS overnight, embedding in Tissue-Tek OCT (Sakura), and freezing at –80°C. Mouse and human organoid sections were prepared as described (18); 6 μm thick sections were cut and stained using standard frozen IHC protocols. The antibodies used are listed in Supplemental Table 3. Images were recorded with a confocal laser-scanning microscope (LSM 710, Carl Zeiss) and processed using ImageJ software (NIH).

### Bulk RNA-Seq and data analysis.

RNA-Seq was performed by the Columbia Genome Center. Poly-A pull-down was used to enrich mRNA from small intestinal epithelial cells sorted from 4- to 6-week-old NFKO or *Ins2*-tomato^^+^^ cells from drug-treated organoids. Libraries were constructed and then sequenced using Illumina NovaSeq 6000. Differentially expressed genes were tested using DESeq2. Pathway enrichment was assessed through the preranked version of GSEA (54).

### scRNA-Seq and data analysis.

Tomato^^+^^ cells were isolated and sorted from NFKO mice as described above. Samples’ viabilities above 90% were processed using the 10× Genomics 3′ Single Cell Gene Expression Microfluidics Platform. Library preparation and sequencing were performed by the Columbia Genome Center as described (55). The R package Seurat was used to do the clustering analysis and cell-type annotation (56) for the raw counts of scRNA-Seq data analysis. The differentiation potential of INS^^+^^ and INS^^neg^^ cells from NFKO mice was predicted using CytoTRACE (26).

### In vitro tissue and organoid insulin secretion assay.

One centimeter of duodenum (after removal of pancreas) or cultured human organoids (after removal of medium and Matrigel) was preincubated in Krebs buffer (2.6 mM glucose) for 1 hour, then switched to stimulation with Krebs buffer (2.6 mM glucose, 16.8 mM glucose or 30 mM KCl) for another hour. Supernatant was collected for the insulin ELISA measurement. Secreted insulin was normalized with total tissue protein.

### Data availability.

The bulk RNA-Seq and scRNA-Seq data were deposited in the NCBI’s Gene Expression Omnibus (GEO GSE201832, GSE213445, and GSE201776).

### Statistics.

Data analysis was conducted using Prism 6.0 software (GraphPad) unless otherwise stated. Proper statistical methods were chosen based on data type and distribution. The statistical test and significance levels are indicated in the figure legends.

### Study approval.

All animal studies were approved and overseen by the Columbia University IACUC (AABG6551). Human small intestine tissue for organoid culture was obtained in accordance with approval by the IRB of Columbia University (AAAS9243). The University of Southern California and Children’s Hospital Los Angeles (Los Angeles, California, USA) IRBs approved all procedures and collection of human fetal tissue samples (HS-19-00837).

## Author contributions

WD designed and executed experiments, analyzed results, and wrote the manuscript. JW analyzed the scRNA-Seq data. T Kuo, LW, WMM, JS, HW, T Kitamoto, YL, RJC, JF, NS, and BSD performed experiments and edited the manuscript. YWC, BHG, and MET collected fetal tissue and performed the human fetal tissue–related experiments. LER and KM performed surgeries from which donor samples were obtained. NG and IB maintained the mouse strain. YL and SB provided FBT compounds in this study. DA designed experiments, oversaw research, and wrote the manuscript.

## Supplementary Material

Supplemental data

## Version 1 10/25/2022

In-Press Preview

## Version 2 12/15/2022

Electronic publication
