# Supplementary material for: Pharmacological conversion of gut epithelial cells into insulin-producing cells lowers glycemia in diabetic animals
Source: J Clin Invest. 2022 Dec 15;132(24):e162720. doi: 10.1172/JCI162720 (PMC9754100; doi:10.1172/JCI162720)
Supplement: Supplemental data [file jci-132-162720-s139.pdf]

Pharmacological conversion of gut epithelial cells into insulin-producing cells lowers glycemia in diabetic animals

Supplementary Information:

This appendix has been provided by the authors to give extended information about the present study.

Figure S1. Insulin staining in human gut and adult pancreas tissues, related to Figure 1

Figure S2. Neurog3-derived canonical 5HT- and lysozyme-immunoreactive cells are $CD24^+$, related to Figure 2

Figure S3. Validation of FoxO1 knockout efficiency of Neurog3 derived cells from NFKO mice, related to Figure 2

Figure S4. Expanded EEC and goblet-Paneth lineages in NFKO by scRNAseq of Neurog3-derived cells, related to Figure 2

Figure S5. iFOXO1 generates 5HT cell-derived $insulin^+$ cells independent of Notch inhibition, related to Figure 3

Figure S6. QPCR analysis showing *Tph1*, *Lyz1* and *Muc2* expression in mGO following treatment with the differentiation cocktail, related to Figure 4

Figure S7. Heatmap comparing expression level of typical islet and gut epithelial marker genes in sorted Tom+ cells from islets vs. differentiated organoids of $INS2\text{-}Tomato^+$ mice, related to Figure 4

Figure S8. Notch and TGFβ inhibitors expand the Neurog3 derive lineage in NFKO mice, related to Figure 5

Figure S9. IHC staining of lysozyme, MUC2 and 5HT after 5 days vehicle or different compounds treatment STZ mice groups, related to Figure 5

Figure S10. Evaluation of residual pancreatic β cells, related to Figure 5

Figure S11. Expansion of other EEC lineages after DBZ and combination treatment, related to Figure 5

Figure S12. Intestinal insulin immunoreactive cells co-stained with other pancreatic β-cell markers, related to Figure 5

Figure S13. IHC staining of lysozyme, MUC2 and 5HT after 5 days vehicle treated NOD mice and pancreatic β cells staining from vehicle and triple inhibitor (iNotch+iTGFβ+iFOXO1)-treated NOD mice, related to Figure 6

Table S1. Mouse strains used in this study

Table S2. Small molecules and growth factors used in study

Table S3. Antibody information

Supplementary Methods

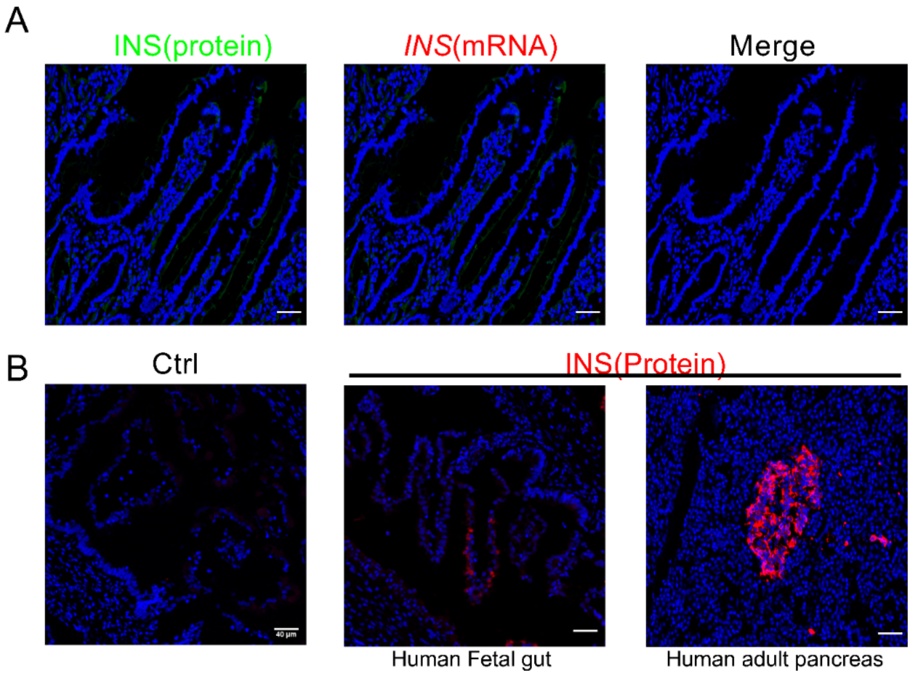

Figure S1. Insulin staining in human gut and adult pancreas tissues

(A) INS-protein (green) and RNAscope (*INS*-mRNA, red) co-staining in adult human duodenum, scale bar = 40µm;

(B) Non-processed image from human fetal gut staining control (no INS primary antibody, only secondary antibody), INS stained human fetal gut and human adult pancreas samples (add both INS primary antibody and secondary antibody), scale bar=40µm, all images were taken with the same setting and the same color scale.

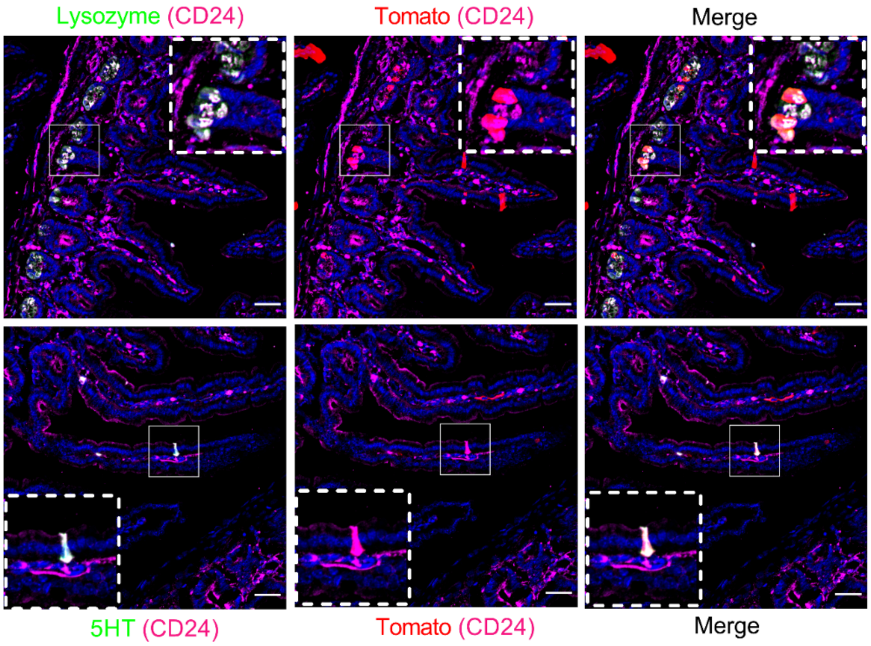

Figure S2. Neurog3-derived canonical 5HT- and lysozyme-immunoreactive cells are $CD24^+$

Immunohistochemistry of Tomato, CD24, Lysozyme (upper panel), or 5HT (lower panel). Lysozyme or 5HT (green), Tomato (red), CD24 (magenta), DAPI (blue), green, red and magenta triple colocalization is shown in white, scale bar: 40um, n=3 mice.

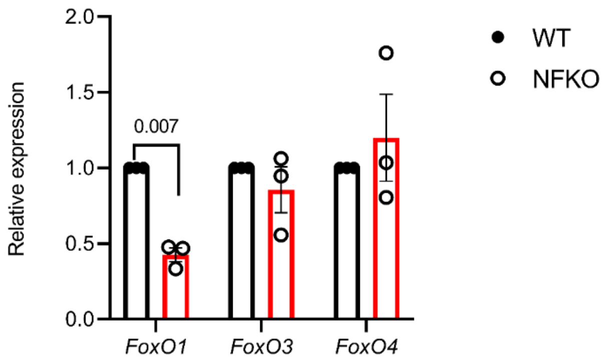

Fig S3. Validation of FoxO1 knockout efficiency of Neurog3 derived cells from NFKO mice.

QPCR of *FoxO1*, *FoxO3* and *FoxO4* expression in sorted Tomato^{+} cells from small intestine of NFKO and WT mice (n=3 independent experiments, bar graphs represent means ± SEM, paired t-test).

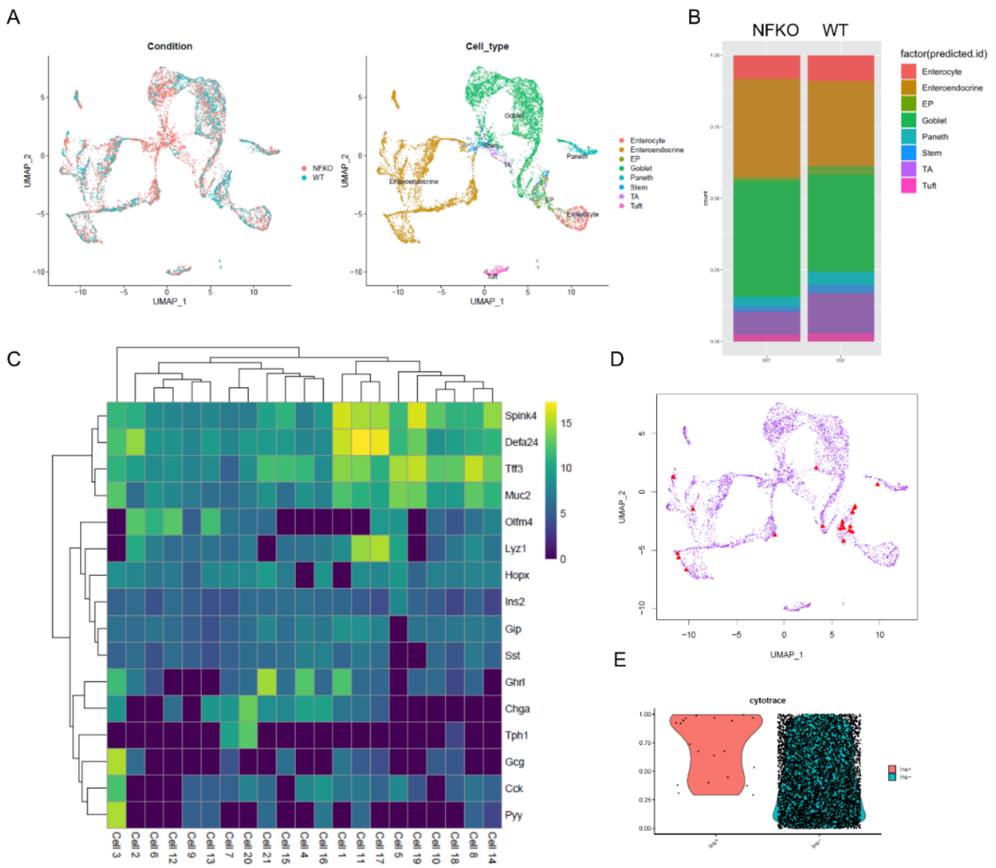

Figure S4. Expanded EEC and goblet-Paneth lineages in NFKO by scRNAseq of Neurog3-derived cells

(A) UMAP plots comparing of NFKO and WT cells clustering (left, sorted from 4 mice) and predicted cell type identities (right);

(B) Quantification of cell-type composition in NFKO and WT *Neurog3*-Tomato cells;

(C) Gene expression heatmap and hierarchical clustering of Insulin^{+} cells in sorted NFKO-Tomato cells;

(D) UMAP plots of Neurog3-Tomato cells from NFKO mice popup of INS expression cells. The Insulin^{+} cells are marked by red triangles;

(E) Violin plots of predicted stemness scores in INS^{+} cell group and INS^{-} cell group. Each cell is visualized as a point. Stemness scores are inferred by CytoTRACE.

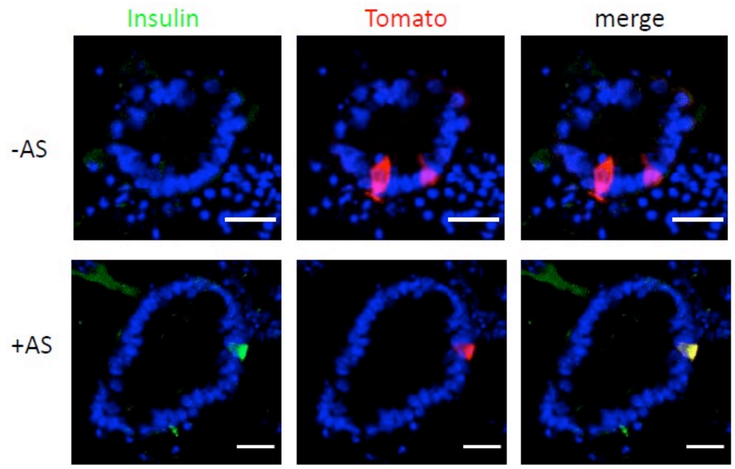

Figure S5. iFOXO1 generates 5HT cell-derived insulin^{+} cells independent of Notch inhibition

Insulin and Tomato double staining of lineage-traced organoids from Tph1Cre^{ERT2} after four days incubation in differentiation (Diff) medium following induction of Tomato reporter with (lower panel) or without (upper panel) iFOXO1 (AS) treatment, Insulin (green), Tomato (red), DAPI (blue), green and red channel colocalization is shown in yellow, n=3 independent experiments, scale bar: 20 µm).

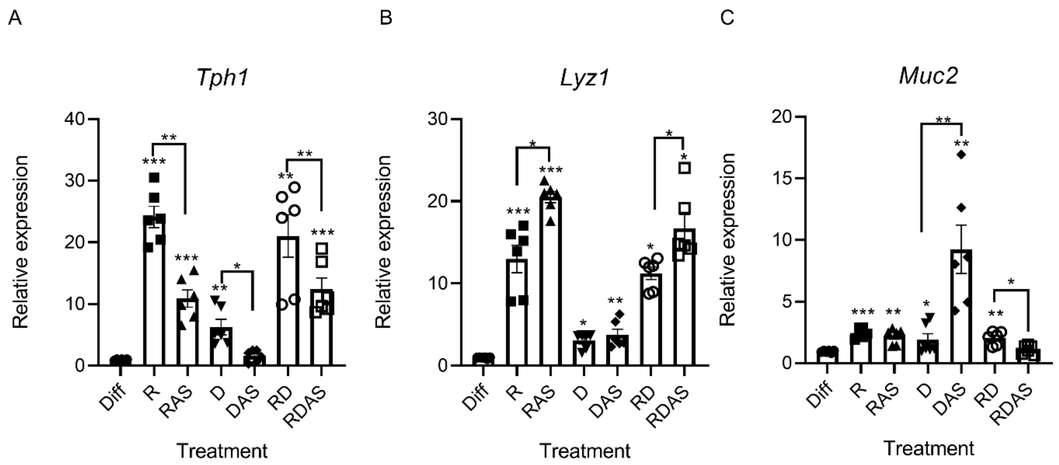

Figure S6. QPCR analysis showing *Tph1, Lyz1* and *Muc2* expression in mGO following treatment with the differentiation cocktail (n=6 independent experiments, bar graphs represent means ± SEM, paired t-test, * P<0.05; ** P<0.01; *** P<0.001).

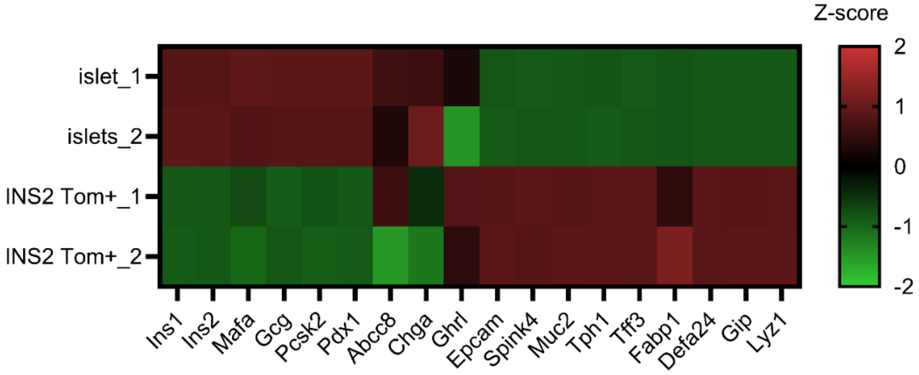

Figure S7. Heatmap comparing expression level of typical islet and gut epithelial marker genes in sorted Tom^{+} cells from islets vs. differentiated organoids of INS2-Tomato mice.

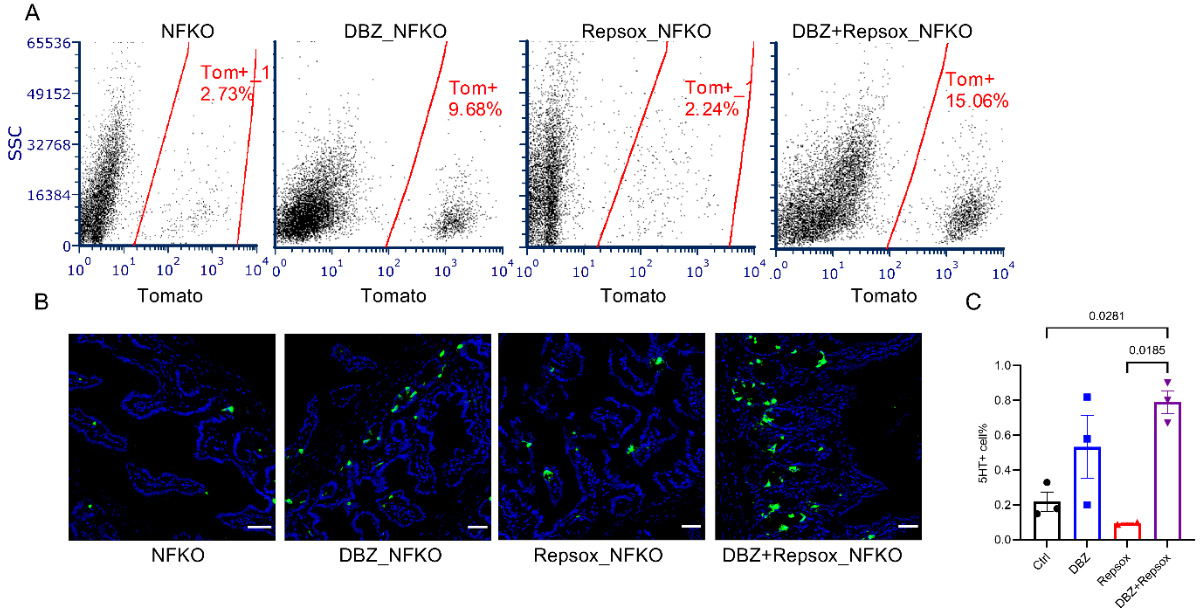

Figure S8. Notch and TGFβ inhibitors expand the Neurog3 derive lineage in NFKO mice

(A) Representative flow cytometry of Tomato^{+} cells sorting from duodenum of Neurog3Cre FoxO1$^{f/f}$;ROSA ^{tdTomato} mice following DBZ, Repsox or combination treatment;

(B) Representative IHC image of 5HT (green) staining in duodenum from Neurog3Cre FoxO1$^{f/f}$; ROSA^{tdTomato} following treatment with DBZ, Repsox or combination, scale bar: 40uM;

(C) Quantitation of 5HT^{+} cells by intracellular staining of FACS sorts in Ctrl, DBZ-, Repsox- or combination-Neurog3Cre FoxO1$^{f/f}$;ROSAtdTomato mice; n=3 mice in each treatment group. Bar graphs represent means ± SEM, two-way ANOVA.

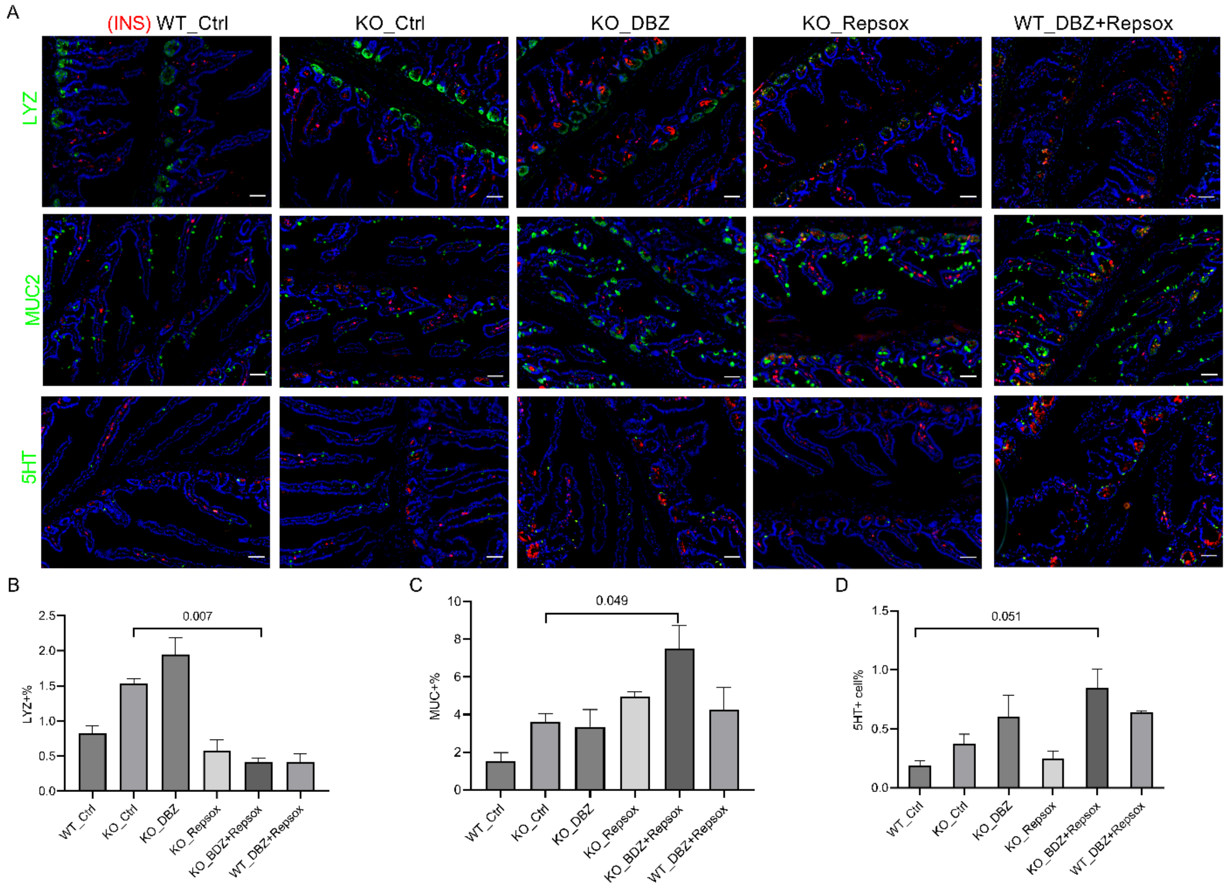

Figure S9. IHC of lysozyme, MUC2 and 5HT after 5-day treatment in STZ mice

(A) Representative IHC image of lysozyme (upper panel, green), MUC2 (middle panel, green) and 5HT (lower panel, green) co-stained with Insulin (red) and DAPI (blue) from 5 different control STZ mice groups, green and red channel colocalization is shown in yellow. Scale bar=40 µm;

(B-D) Quantification of Lysozyme-, MUC2- and 5HT-positive cells percentage (out of total small intestinal cells) from 6 different treatment STZ mice groups, n=3 mice in each group, bar graphs indicate mean± SEM.

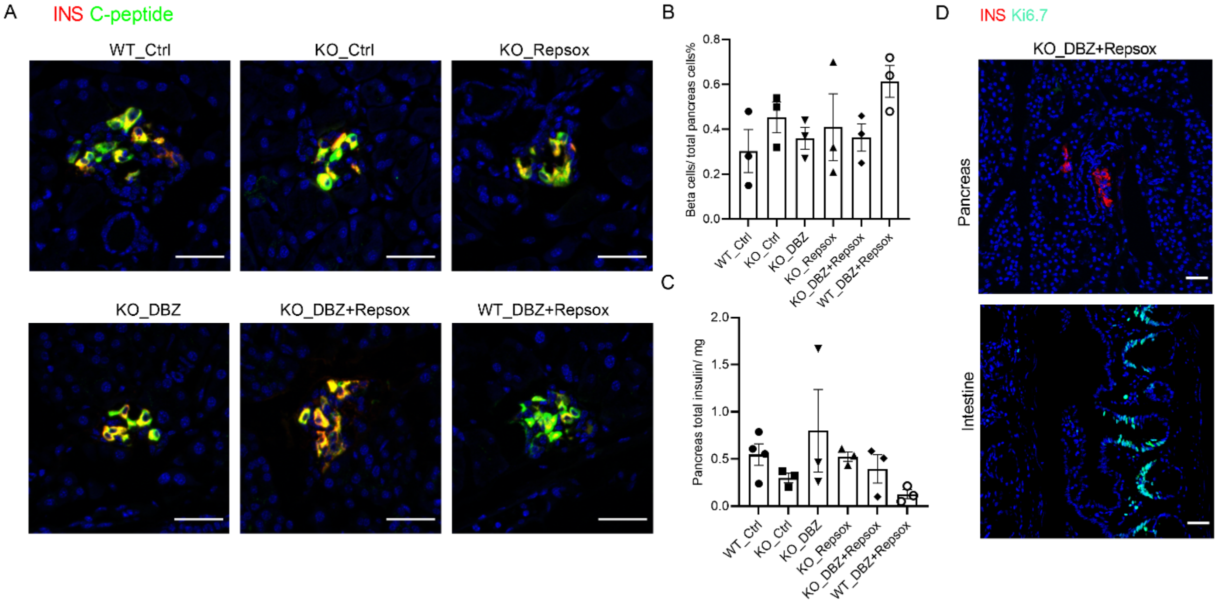

Figure S10. Evaluation of residual pancreatic β-cells

(A) Representative IHC of islet with Insulin (red), c-peptide (green) and DAPI (blue) from 6 different treatment STZ mice pancreatic sections, scale bar=40 µm;

(B) IHC-based whole-pancreas islet quantification. β-cell percentage was calculated from ratio of total INS^+ C-$peptide^+$ cells to total pancreatic cells;

(C) Total insulin measurement from 6 different treatment STZ mice whole pancreas, n=3 or 4 mice in each group, bar graphs indicate mean± SEM.

(D) Representative IHC image of islets and small intestine staining with Insulin (red) and Ki6.7 from DBZ and Repsox combination treatment mice, scale bar=40 µm.

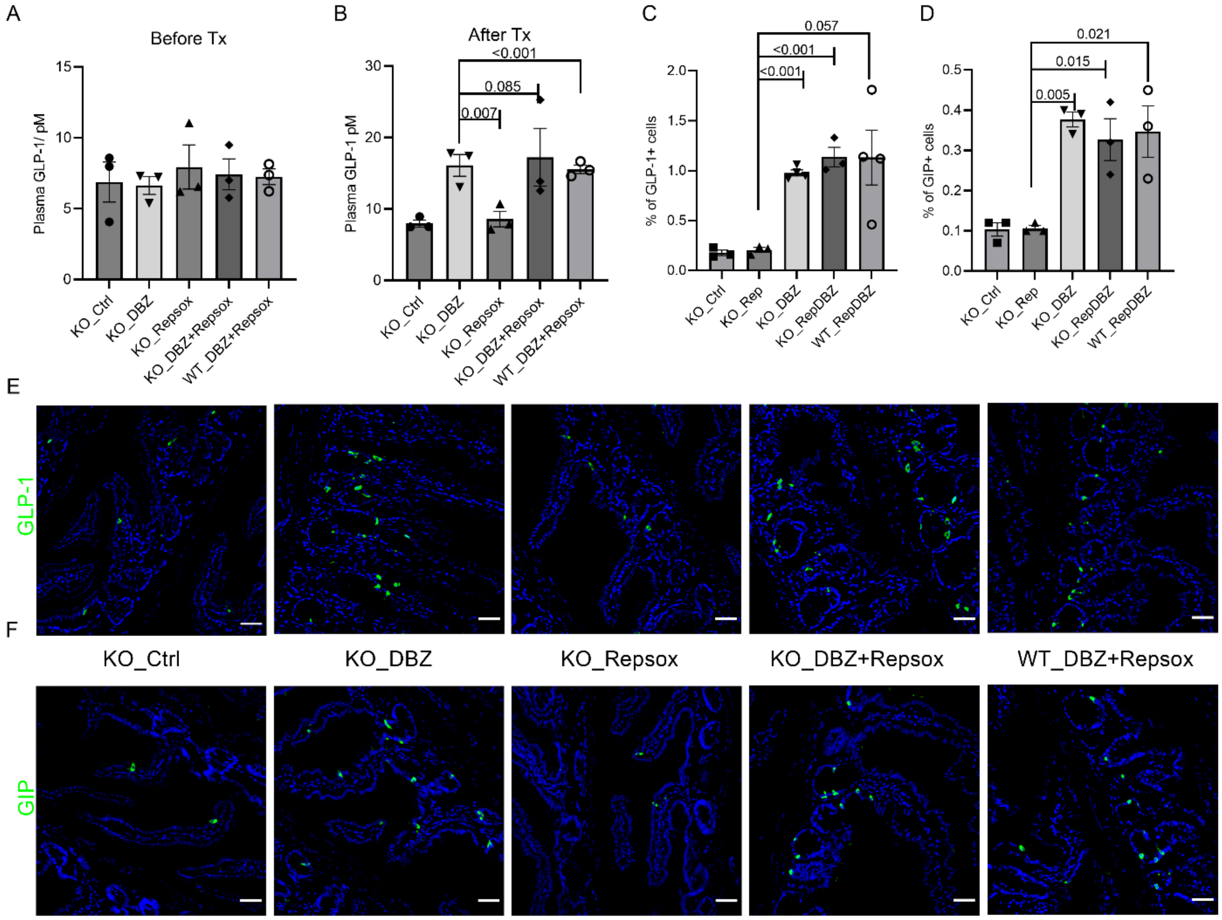

Figure S11. Expansion of other EEC lineages after DBZ and combination treatment

(A) Plasma GLP-1 level before and after (B) 5 different treatment, n=3-4 mice in each treatment;

(C) Quantification of GLP-1 positive cells out of total small intestinal cells, bar graphs indicate mean± SEM, n=3-4 mice in each treatment;

(D) Quantification of GIP positive cells out of total small intestinal cells, bar graphs indicate mean± SEM, n=3-4 mice in each different treatment;

(E-F) Representative IHC image of GLP-1 (E, green) or GIP (F, green) and DAPI (blue) from 5 different treatment STZ mice small intestine, scale bar=40 μm.

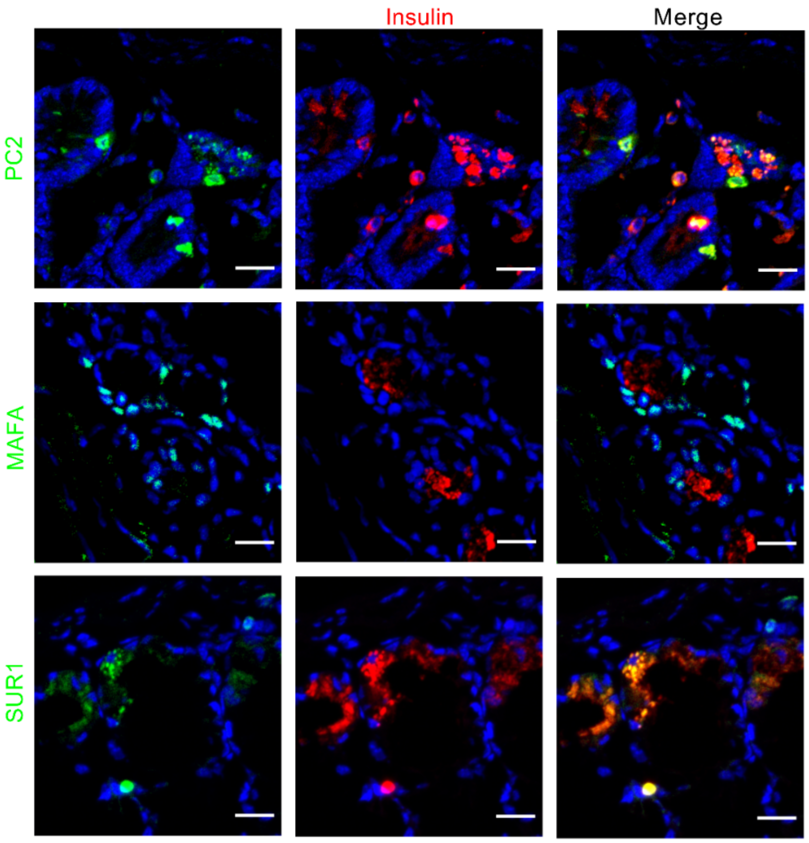

Figure S12. Intestinal insulin immunoreactive cells co-stained with other pancreatic β-cell markers. Representative IHC image of PC2 (upper panel, green), MAFA (middle panel, green) and SUR1 (lower panel, green) co-stained with Insulin (red) in combination therapy-treated STZ-NFKO mice, scale bar=20μm, green and red channels colocalization shown in yellow.

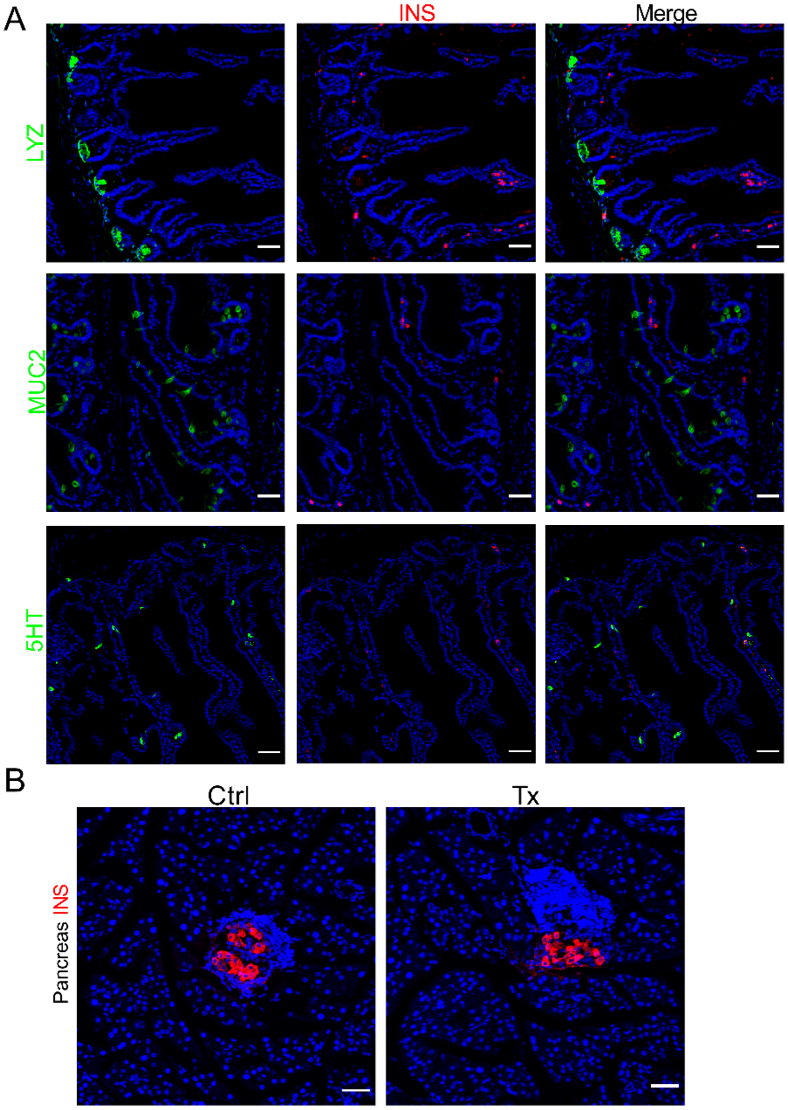

FigS13. Gut and pancreatic IHC in vehicle- or combination-treated NOD mice

(A) Representative IHC image of lysozyme (upper panel, green), MUC2 (meddle panel, green) and 5HT (lower panel, green) co-stained with Insulin (red) and DAPI (blue) from vehicle treated NOD mice (Ctrl), scale bar=40 µm;

(B) Representative IHC image of Insulin^{+} (red) β cells from vehicle and triple inhibitor treated NOD pancreas, DAPI (blue), scale bar=40 µm.

Table S1. Mouse strains used in this study

Mice strain		
Mouse: Tg(Neurog3-cre)C1Able/J	The Jackson Laboratory	JAX: 005667
Mouse: B6.Cg-Gt(ROSA)26$^{Sortm9(CAG-tdTomato)Hze}$/J	The Jackson Laboratory	JAX: 007909
Mouse: NOD/ShiLtJ	The Jackson Laboratory	JAX:001976
Mouse: Tg(Ins2-cre)23Herr	Kuo et al., 2019	N/A
Mouse: FoxO1 $^{fl/fl}$	Matsumoto et al., 2007	N/A
Mouse: Tph1^{CreERT2}	gifted by Prof. Juanita L. Merchant of University of Arizona	N/A
Mouse: Lyz1^{CreER}	Yu et al., 2018	N/A

Table S2. Small molecules and growth factors used in study

Name	Abbreviation	Working Concentration	Company	Cat#
EGF	E	50 ng/mL	R&D	236-EG-200
Noggin	N	Conditional Medium	Gifted by C. J. Kuo, Stanford University, CA	
R-Spondin	Rspo	Conditional Medium	Gifted by H. Clevers, Hubrecht Institute, Netherlands	
Vaproic Acid Sodiumm Salt	V	2mM	Sigma	P4543
DAPT	D	5μM	Sigma	D5942
CHIR99021	C	5μM	Tocris Bioscience	4423
Repsox	R	5μM	Selleckchem	E-616452
IWP2	Iw	10 μM	Sigma	I0536
(Z)-4-Hydroxytamoxifen	4-OH-TAM	0.5 μM	Sigma	H7904
AS 1842856	AS	300nM	Tocris Bioscience	4265
ISX-9	Is	40 μM	Tocris Bioscience	4439
PD0325901	Pd	1μM	Sigma	PZ0162
5-Aza-2′-deoxycytidine	Adc	500nM	Sigma	A3656
thyroid hormone	T3	1μM	Sigma	T6397
Y-27632	Y	1μM	Sigma	Y0503
dibenzazepine (DBZ)			Apexbio	A4018
FBT 10		1μM	ForkheadBio Therapeutics	
FBT 374		1μM	ForkheadBio Therapeutics	
PF-03084014	PF		Selleckchem	S8018

All drugs were dissolved in DMSO and diluted to the indicated concentration with medium during the in vitro treatment.

Table S3. Antibody information

Antibody information			
Name	Company	Cat.No	dilution
FOXO1	Abcam	ab39670	1:100
GLP-1	Abcam	ab22625	1:500
5HT	Abcam	ab66047	1:500
GIP	ABBIOTEC	250668	1:500
Ki6.7	Abcam	ab15580	1:500
RFP	Rockland	600-401-379	1:100
RFP antibody [5F8]	Chromotech	5f8-20	1:500
Insulin	Dako	A0564	1:200
Insulin	Biorad	5330-0104G	1:200
C-peptide	Thermo	PA5-85595	1:100
MUC2	Abcam	ab272692	1:300
Lysozyme	Dako	A0099	1:100
SUR1	Abcam	ab32844	1:50
MafA	Abcam	ab26405	1:100
PC2	Thermofisher	PA1-058	1:200
CD326 (EpCAM) Monoclonal Antibody (G8.8), FITC	Thermofisher	11-5791-82	1:500
CD24 Monoclonal Antibody (M1/69), APC	Thermofisher	17-0242-82	1:100
Donkey anti-goat-Alexa488	Invitrogen	A11055	1:1000
Donkey anti-rabbit-Alexa555	Invitrogen	A31572	1:1000
Goat anti-Rabbit-Alexa488	Invitrogen	A11008	1:1000
Goat anti-Mouse-Alexa488	Invitrogen	A11029	1:1000
Goat anti-Mouse-Alexa555	Invitrogen	A21424	1:1000

Supplementary methods

Animal studies

Tg(Neurog3-cre)C1Able/J (#005667, Ngn3-Cre)[1] and B6.Cg-Gt(ROSA)26$^{Sortm9(CAG-tdTomato)Hze}$/J (#007909) were from the Jackson Laboratories. Tg(Ins2-cre)23Herr [2] and FoxO1 $^{fl/fl}$ mice [3] have been described. Tph1^{CreERT2} mice were kindly gifted by Prof. Juanita L. Merchant of University of Arizona and Lyz1^{CreER} mice by Prof. Nan Gao of Rutgers University [4]. Mice were housed in 12h light/dark cycle (7AM/7PM) barrier facility with free access to water and food. All animal studies were approved by and overseen by Columbia University Institutional Animal Care and Use Committee (IACUC).

Physiological Studies

A single injection of Streptozotocin (STZ, 170 mg/kg, S0130, Sigma, St.Louis, MO) was administrated intraperitoneally to induce diabetes in 6 to 8-week old male NFKO and littermate male FoxO1 $^{f/f}$ (WT) mice upon High-Dose STZ Induction Protocol from Animal Models of Diabetic Complications Consortium (AMDCC) (http://www.AMDCC.org). Ad libitum blood glucose levels and body weight were monitored every 2-3 days after STZ injection. Tail blood glucose after 4h of fasting was measured on days 0 and 7 using Contour Next glucometer. Mice that were not hyperglycemic (Fast Blood Glucose > 250 mg/dl) 7 days after STZ administration were excluded from further study. Mice were randomized base on blood glucose and body weight.

For in vivo drug treatment, mice were injected intraperitoneally with 25mg/kg DBZ once per day for 2 days and gavaged with 10mg/kg Repsox once per day for 5 to 7 days. Weight and ad libitum glucose levels were monitored every 2 days during treatment. For NOD mice in vivo drug treatment, FBT10, PF-03084014 and Repsox were dosed orally twice daily at 50 mg/kg/dose, 150 mg/kg/dose and 10mg/kg/dose, respectively. In the fasting-refeeding study, mice were fasted for 4 hours followed by 1 hour refeed. Blood was collected from tail vein with DPP4 inhibitor and plasma insulin or GLP-1 were

measured by insulin ELISA kit (Mercodia, Uppsala, Sweden) or GLP-1 ELISA Kit (Crystal Chem, Elk Grove Village, IL). In oral glucose tolerant test (OGTT), mice were fasted for 4 hours followed by gavaging of 2 g/kg (10 ml/kg) of D-glucose (Sigma) dissolved in distilled water. Glucose was measured at 0, 15, 30, 60 and 120 min by tail vein sampling.

Human Tissues

Surgically resected intestinal tissues or endoscopic biopsy samples were obtained from 8 patients from the Columbia University Irving Medical Center /Presbyterian Hospital and Vanderbilt Clinic. The institutional review boards at Columbia University have approved all procedures and all samples were obtained with informed consent. All human fetal tissue samples were collected under Institutional Review Board Approval at both the University of Southern California and Children's Hospital Los Angeles. Consent for tissue donation was obtained after the patient had already made the decision for pregnancy termination by Dilation and Curettage or Dilation and Evacuation and was obtained by a different clinical staff member than the physician performing the procedure. All tissue was de-identified, and the only clinical information collected was gestational age and the presence of any material of fetal diagnoses. Intestinal samples ranging in age from 15 to 17 weeks of gestation were received immediately after elective terminations and fixed in 4% paraformaldehyde, dehydrated with 30% sucrose, processed for OCT embedding, followed by sectioning and immunostaining.

Chemicals

All small molecule information for intestinal treatment is listed in Table S2. DBZ was from Apexbio Technology; RepSox and PF-03084014 were from Selleck Chemical; FBT10 from ForkheadBio Therapeutics. For STZ mice in vivo treatment, DBZ and Repsox were formulated in 1% DMSO, 0.5% methylcellulose and 0.2% Tween-80 PBS solution, respectively. For NOD mice in vivo treatment, FBT10,

PF-03084014 and Repsox was formulated together into N,N-Dimethylacetamide: Solutol HS 15: water= 5:10:85 (v/v/v) solution, pH4-5.

Gut Organoid Cultures

For mouse small intestinal organoids, crypts were isolated using EDTA chelation from the duodenum as described [5] and cultured in complete growth medium that included advanced Dulbecco's modified Eagle's medium and F12, DMEM/F12 (Gibco, Grand Island, NY) containing penicillin or streptomycin 100 U/mL, HEPES 10 mmol/L, Glutamax 2 mmol/L, B27 supplements (Gibico), murine epidermal growth factor (mEGF) 50 ng/mL (Peprotech, East Windsor, NJ), N-acetylcysteine 1 mmol/L (Sigma), R-spondin-1 (conditioned medium 1ml/50ml, gifted by Prof. Calvin J. Kuo, Stanford University, CA), Noggin (conditioned medium 1ml/50ml, gifted by Prof. Hans Clevers, Hubrecht Institute, Netherlands). Differentiation medium consisted of the same components as complete growth medium without R-spondin-1, Noggin and hEGF.

For human small intestinal organoids, crypts were isolated using EDTA chelation from the duodenum as previously described [6]. IntestiCult™ Organoid Growth Medium (Human) or IntestiCult™ Organoid Differentiation Medium (Human) were used for culture or differentiation of human gut organoids (STEMCELL Technologies, Vancouver, Canada). Organoids were used prior to passage 3 for optimal efficiency of EEC and β-like cell induction.

Intestinal epithelial cell isolation and sorting

4- to 6-week-old NFKO mice were used to isolate single intestinal cell preparations as described [7]. A segment of the proximal intestine representing a 5-cm region from 2 to 7 cm distal to the pyloric sphincter was used for cell isolation. Attached pancreata were carefully removed under a dissection microscope to avoid pancreatic β-cell contamination. Isolated intestinal epithelial cells (10^7 cells/1ml)

were incubated with Mouse BD Fc Block™ (55314, BD, Franklin Lakes, NJ) for 10 minutes on ice in IESC media (DMEM/F12, B27, Glutamax, Penicillin/Streptomycin, 500 mM N-acetyl-cysteine and 10µM Y27632). Cells were stained 20 min with APC-conjugated anti-CD24 antibody (1:100 dilution, 138506, Biolegend, San Diego, CA) and FITC-conjugated anti-Epcam antibody (1:500 dilution, 118208, Biolegend), washed twice with IESC media and DAPI (0.1 µg/ml, D1306, Invitrogen, Waltham, MA) was added prior to sorting using BD Influx.

Flow cytometric analysis of epithelial cells

Single cell suspension was obtained by enzymatic digestion of intestinal mucosa or cultured organoids [7,8]. Suspended cells were first stained with live/dead cell staining kit (Invitrogen, L34964), then fixed in BD Cytofix™ fixation buffer (BD, 554655) for 20 min. Cells were washed in permeabilization buffer (0.2% Saponin, 0.5% BSA and 2mM EDTA in PBS) twice followed by intracellular primary and secondary antibody staining. After 3 washes with permeabilization buffer, cells were resuspended in FACS buffer (0.5%BSA and 2mMEDTA in PBS) for sorting or FACS analysis. When sorted cells were used for RNA isolation, 0.2% RNaseOUT (Invitrogen, 10777-019) was added to the antibody incubation and FACS buffer before sorting.

RNA isolation and quantitative PCR analysis

Cultured gut organoids or FACS sorted cell were in 1ml TRIzol (ThermoFisher, Waltham, MA). RNA was isolated using RNeasy mini kit or RNeasy Micro kit (Qiagen, Germantown, MD) followed by reverse transcription using qScript cDNA Synthesis Kit (QuantaBio, Beverly, MA). RNA isolation from intercellular stained cell sample was as described [9]. QPCR was performed with GoTaq® qPCR Master Mix (Promega, Madison, WI). QPCR primers sequence will be provided upon request. Gene expression levels were

normalized to HPRT (hypoxanthine phosphoribosyl transferase) using the $2^{-\Delta\Delta Ct}$ method and are presented as relative transcript levels.

Quantitative measurement of conversion insulin-producing cells with cultured organoids

Primary gut crypts which derived from a mouse bearing an Rip-Cre;Rosa26^{tdTomato} reporter allele placed in culture, and then induced to undergo cell conversion by applying a protocol based on published patent US20170349884A1. Briefly, the first 2 days after seeding the organoids, crypts cultured in complete growth medium with CHIR99021(5µM), Valproic acid (2mM) and Y-27632(10µM) to enhance the stem cell vitality. At Day3, DAPT (5µM), Repsox (5µM), ISX-9 (40 µM) and Adc (500nM) were added to complete growth medium. At Day 4-5, organoids were treated with differentiation medium supplemented with DAPT (5µM), Repsox (5µM), ISX-9 (40 µM), Adc (500nM), and IWP-2(10µM) to induce cell conversion. At Day 6, organoids were incubated in differentiation medium with DAPT (5µM), Repsox (5µM), PD0325901 (1µM), T3 (1µM), CHIR 99021 (5µM) to induce further maturation of β-like cells. During the differentiation process (Day 4-6), a FOXO1 inhibitor (e.g., AS or one of the FBT compounds) was added to the medium along with the inhibitor cocktails listed above. Thereafter, cells were analyzed by flow cytometry, and cells in which *Ins2* expression has been activated were sorted from other epithelial cells by flow cytometry. These cells were collected by flow, RNA extracted and analyzed for expression of β-cell-specific genes. To evaluate the efficiency of cell conversion to INS2-positive cells we calculated a Tom Score by multiplying INS2 tomato intensity, percentage of INS2 Tomato cells, and percentage of live cells.

In Situ Hybridization by RNAscope

RNAscope was performed using the RNAscope® 2.5 HD Detection Reagent – RED kit (Advanced Cell Diagnostics, Hayward, California) combined with immunofluorescence according to manufacturer's

instructions. A human insulin probe (ACD catalogue number 313571, NCBI reference sequence NM_000207.2) was used to detected insulin mRNA.

Immunohistochemistry

Small intestine was isolated from 6 to 8-week-old or drug-treated mice. After harvesting, the tissues were rinsed with PBS, coiled into Swiss rolls, and fixed in 4% PFA for 2 hours, followed by dehydration in 30% sucrose in PBS overnight, embedding in Tissue-Tek O.C.T (Sakura, Torrance, CA), and freezing at –80°C. Mouse and human organoids sections were prepared as described [10]. 6μm-thick sections were cut and stained with standard frozen-IHC protocols.

Tile scanning was used in insulin+ cells analysis from the human fetal small intestine roll. ¼ roll was scanned and INS-mRNA+ and INS protein+ cells were counted manually. For FOXO1 and Insulin costain experiment, we only took images from fields that contain insulin protein-positive staining cells. We averaged technical replicates within each given donor into a single data point, showing only 3 data points in each bar graph. The primary and secondary antibodies used are listed in Table S2. Images were recorded with a confocal laser-scanning microscope (LSM 710, Carl Zeiss, NY) and processed using Image J software (National Institutes of Health).

Insulin secretion assay

1 well of 24-well plate cultured hGO (completely remove matrigel) or ~1 cm duodenal segments (completely remove pancreata) were washed 3 times with cold PBS. Pre-incubate in 2.8mM Glucose - Kreb buffer (118.5 mM NaCl, 2.54mM $CaCl_2$, 1.19mM KH_2PO_4, 1.19mM $MgSO_4$, 10mM HEPES, and 2% BSA, PH=7.4) at 37°C for 1hour, take out the buffer, add 200ul 2.8mM Kreb buffer, incubate at 37°C for 1 hour to collect basal insulin samples. Followed by taking out the basal samples, 200ul simulation buffer (16.8mM Glucose-Kreb buffer or 30mM KCl-Kreb buffer) was added for another 1-hour

incubation at 37°C for the collection of stimulated insulin samples. hGOs or duodenal segments were washed with cold PBS twice to get rid of BSA, and then lysed in RIPA buffer with protease inhibitors for insulin content or total protein measurement. Insulin was determined by human or mouse insulin ELISA (Mercodia).

Bulk RNA-sequencing and data analysis

RNA-sequencing was performed by the Columbia Genome Center. Poly-A pull-down was used to enrich mRNA from small intestinal epithelial cells sorted from 4 to 6-week-old NFKO or Ins2-tomato$^+$ cells from drug-treated mice. Library construction was done using Illumina TruSeq chemistry (intestinal epithelial cells) or the Clontech Ultra Low v4 kit for cDNA synthesis followed by NexteraXT (sorted cells from organoids). Libraries were sequenced using Illumina NovaSeq 6000. Samples were multiplexed in each lane, yielding targeted numbers of paired-end 100bp reads for each sample. RTA (Illumina) was used for base calling and bcl2fastq2 (version 2.19) for converting BCL to fastq format, coupled with adaptor trimming. A pseudoalignment to a kallisto index was created from transcriptomes (Mouse: GRCm38) using kallisto (0.44.0). Differentially expressed genes were tested under various conditions using Sleuth or DESeq2, R packages designed to test differential expression between two experimental groups from RNA-seq counts data. Pathway enrichment was assessed through the pre-ranked version of Gene Set Enrichment Analysis (GSEA) [11].

ScRNA-seq and data analysis

Tomato$^+$ cells were isolated and sorted from NFKO mice as described above. Viability >90% was obtained for all samples as determined by trypan blue staining. 10x Genomics 3' Single Cell Gene Expression (GEX) microfluidic cell processing, library preparation and sequencing were performed by the Columbia Genome Center as described [12]. The raw counts of scRNA-seq data were analyzed as follow.

The R package Seurat was used to do the clustering analysis and cell type annotation. [13]. For the quality control, cells with either the percentage of reads mapped to the mitochondrial genome higher than 15%, or total UMI counts higher than 1e-6, or number of feature RNA smaller than 200 were removed. The "LogNormalize" normalization method was used to normalize library size. Clustering analysis was done using the unsupervised graph-based function of the Seurat package. First, the highly variable genes were identified, and data scaled. Then principal component analysis (PCA) was performed on the scaled data using highly variable genes. The neighbors of cells were found based on the first 50 PCs. The modularity was optimized by the Louvain algorithm. Finally, the clustering results are visualized on PCA plot. The integration method in Seurat was used for annotating the cell types based on known cell types in reference dataset [14]. Single-cell transcriptome data of mouse small intestinal epithelium was obtained from GSE92332 and used as reference [15]. The differentiation potential of INS^{+} and INS^{neg} cells from NFKO mice was predicted using CytoTRACE [16].

Statistical analysis

Data analysis was conducted using Prism 6.0 software (GraphPad Software, San Diego, California) unless otherwise stated. Sample sizes were determined by the reproducibility of the experiments and are similar to those generally employed in the field. Statistical significance in two-way comparisons was determined by a Student's t-test or paired t-test when data met the normal distribution, whereas ANOVA was used when comparing more than two datasets. If the data did not meet the normal distribution, Mann-Whitney test were used. The statistical test and significance level are indicated in the figure legends.

Reference

1. Schonhoff SE, Giel-Moloney M, Leiter AB. Neurogenin 3-expressing progenitor cells in the gastrointestinal tract differentiate into both endocrine and non-endocrine cell types. Dev Biol 2004;270:443-54.
2. Kuo T, Kraakman MJ, Damle M, et al. Identification of C2CD4A as a human diabetes susceptibility gene with a role in beta cell insulin secretion. Proc Natl Acad Sci U S A 2019;116:20033-20042.
3. Matsumoto M, Pocai A, Rossetti L, et al. Impaired regulation of hepatic glucose production in mice lacking the forkhead transcription factor Foxo1 in liver. Cell Metab 2007;6:208-16.
4. Yu S, Tong K, Zhao Y, et al. Paneth Cell Multipotency Induced by Notch Activation following Injury. Cell Stem Cell 2018;23:46-59 e5.
5. Sato T, Clevers H. Primary mouse small intestinal epithelial cell cultures. Methods Mol Biol 2013;945:319-28.
6. Fujii M, Matano M, Nanki K, et al. Efficient genetic engineering of human intestinal organoids using electroporation. Nat Protoc 2015;10:1474-85.
7. Magness ST, Puthoff BJ, Crissey MA, et al. A multicenter study to standardize reporting and analyses of fluorescence-activated cell-sorted murine intestinal epithelial cells. Am J Physiol Gastrointest Liver Physiol 2013;305:G542-51.
8. Sato T, Stange DE, Ferrante M, et al. Long-term expansion of epithelial organoids from human colon, adenoma, adenocarcinoma, and Barrett's epithelium. Gastroenterology 2011;141:1762-72.
9. Thomsen ER, Mich JK, Yao Z, et al. Fixed single-cell transcriptomic characterization of human radial glial diversity. Nat Methods 2016;13:87-93.

10. Bouchi R, Foo KS, Hua H, et al. FOXO1 inhibition yields functional insulin-producing cells in human gut organoid cultures. Nat Commun 2014;5:4242.

11. Subramanian A, Tamayo P, Mootha VK, et al. Gene set enrichment analysis: a knowledge-based approach for interpreting genome-wide expression profiles. Proc Natl Acad Sci U S A 2005;102:15545-50.

12. Capdevila C, Calderon RI, Bush EC, et al. Single-Cell Transcriptional Profiling of the Intestinal Epithelium. Methods Mol Biol 2020;2171:129-153.

13. Satija R, Farrell JA, Gennert D, et al. Spatial reconstruction of single-cell gene expression data. Nat Biotechnol 2015;33:495-502.

14. Stuart T, Butler A, Hoffman P, et al. Comprehensive Integration of Single-Cell Data. Cell 2019;177:1888-1902 e21.

15. Haber AL, Biton M, Rogel N, et al. A single-cell survey of the small intestinal epithelium. Nature 2017;551:333-339.

16. Gulati GS, Sikandar SS, Wesche DJ, et al. Single-cell transcriptional diversity is a hallmark of developmental potential. Science 2020;367:405-411.
